# Mapping the arterial vascular network in an intact human kidney using hierarchical phase-contrast tomography

**DOI:** 10.1038/s44303-025-00090-2

**Published:** 2025-09-03

**Authors:** Shahrokh Rahmani, Daniyal J. Jafree, Peter D. Lee, Paul Tafforeau, Joseph Brunet, Sonal Nandanwar, Yang Zhou, Joseph Jacob, Alexandre Bellier, Maximilian Ackermann, Danny D. Jonigk, Rebecca J. Shipley, David A. Long, Claire L. Walsh

**Affiliations:** 1 Department of Mechanical Engineering, University College London, London, UK; 2 National Heart & Lung Institute, Faculty of Medicine, Imperial College London, London, United Kingdom; 3 Developmental Biology and Cancer Research & Teaching Department, UCL Great Ormond Street Institute of Child Health, University College London, London, UK; 4 Wellcome Trust Sanger Institute, Hinxton, UK; 5 UCL Centre of Kidney and Bladder Health, UCL, London, UK; 6 European Synchrotron Radiation Facility, Grenoble, France; 7 Satsuma Lab, Centre for Medical Image Computing, UCL, London, UK; 8 Lungs for Living Research Centre, UCL, London, UK; 9 Department of Anatomy (LADAF), Grenoble Alpes University, Grenoble, France; 10 Institute of Anatomy, University Medical Center of the Johannes Gutenberg University Mainz, Mainz, Germany; 11 Institute of Pathology and Department of Molecular Pathology, Helios University Clinic Wuppertal, University of Witten-Herdecke, Wuppertal, Germany; 12 Institute of Pathology, RWTH Aachen Medical University, Aachen, Germany; 13 German Center for Lung Research (DZL), Biomedical Research in Endstage and Obstructive Lung Disease Hannover (BREATH), Hannover, Germany

**Keywords:** Engineering, Kidney diseases

## Abstract

The architecture of kidney vasculature is essential the organ's specialised functions, yet is challenging to structurally map in an intact human organ. Here, we combined hierarchical phase-contrast tomography (HiP-CT) with topology network analysis to enable quantitative assessment of the intact human kidney vasculature, from the renal artery to interlobular arteries. Comparison with kidney vascular maps described for rodents revealed similar topologies to human, but human kidney vasculature possessed a significantly sharper decrease in radius from hilum to cortex, deviating from theoretically optimal flow resistance for smaller vessels. Structural differences in kidney hilar, medullary and cortical vasculature reflected unique functional adaptations of each zone. This work represents the first time the arterial vasculature of an intact human kidney has been mapped beyond segmental arteries, potentiating novel computational models of kidney vascular flow in humans. Our analyses have implications for understanding how blood vessel structure collectively scales to facilitate specialised functions in human organs.

## Introduction

The vasculature of the kidney is highly specialised and serves multiple functions, including the delivery of oxygen and nutrients to the organ’s parenchyma, whilst also facilitating plasma ultrafiltration and solute reabsorption. Despite only comprising approximately 1% of body weight, the kidney receives up to 20% of cardiac output^1^. Blood enters the kidney through the renal artery, which branches from the abdominal aorta and enters the kidney hilum. Once within the kidney, the renal artery divides hierarchically, first into segmental or renal feeding arteries which pass through the kidney pelvis, then branching into interlobar arteries which pass through columns between the pyramids of the kidney medulla. At the distal end of the kidney columns, interlobar arteries branch into arcuate arteries that arch around the outer surface of the kidney pyramids. From these, the interlobular vessels branch and penetrate the surrounding kidney cortex, before finally terminating at afferent arterioles^1^. This complex network perfuses specialised capillary networks, including glomerular capillaries across which plasma ultrafiltration occurs, efferent arterioles and peritubular capillaries or vasa recta, which facilitate dynamic solute exchange in the cortex and medulla, respectively. Thereafter, venous return follows the arterial supply out of the organ^2^.

Structural and molecular changes to the kidney vasculature are a common feature of kidney pathologies, including multiple aetiologies of chronic kidney disease (CKD) and transplant rejection in both animal models and patients^3^. Therefore, studying kidney vascular patterning has implications for understanding the basis of kidney function in health and disease, and aids surgical planning for tumour resection, nephrectomy and transplantation. Vascular geometries also have a central role to play in computational models that underpin the creation of digital twins, such as through the generation of synthetic data^4^, and blood flow modelling^4–9^, which are playing an increasing role in biomedical research.

Vascular imaging of the kidney has advanced following technological innovations in micro-computed tomography (μCT)^9–11^, magnetic resonance imaging (MRI) and μMRI^12,13^, ultrasound^14^, lightsheet microscopy^15,16^ and photoacoustic imaging^17–19^. These techniques have been used to generate quantitative analyses of vascular network geometry in intact kidneys of model organisms, particularly rodents, in which kidney diameter reaches up to 12 mm^20^. Comparatively human kidneys, with a diameter of approximately 5 cm^21^ are far more challenging to image at high resolution whilst still intact. Corrosion casting of human kidneys has highlighted vascular heterogeneity and generated intricate 3D casts (down to 100 µm) but provides limited quantitative or accessible digitised geometries of the vascular network^22^. Optical clearing and lightsheet microscopy have been used to quantify portions of the human kidney vascular network^23^. However, as far as the authors are aware, there is no published dataset capturing the intact vascular network of the human kidney beyond approximately six vessel divisions without physical sectioning or subsampling of the tissue^24^. MRI has been used to quantify larger vessels both in vivo and post mortem^25,26^, but for large volumes of interest (VOI), lacks the resolution capable of imaging small vessels and arterioles^25^. µMRI can be used to image down to ~50 µm/voxel, but is limited to smaller biological samples such as rodent kidneys^13^. CT and µCT have been used extensively to image and analyse rodent renal vasculature^9–11^, and have also been applied to study the vasculature of ex vivo human lung^27^ and fetal kidney. However, no detailed segmentation and quantitative analysis of vascular networks in the human kidney have been performed down to the level of arterioles, because of a lack of available imaging data.

Due to this limitation, analysis of human kidney vascular networks is often focused on the first three, large branches of the arterial tree^25^, or limited to subregions within the network^28^. Where multiscale modelling has been performed, parameters from rodent kidneys are assumed to be representative of human kidney vascular networks^4,7,9^. However, semi-quantitative studies of human kidney vascular casts have shown large anatomical variation in segmental artery patterning^24^, whilst smaller vessels such as the arcuate arteries, interlobular arteries and afferent or efferent arterioles have not been assessed quantitatively at the organ scale.

**Fig. 1:**
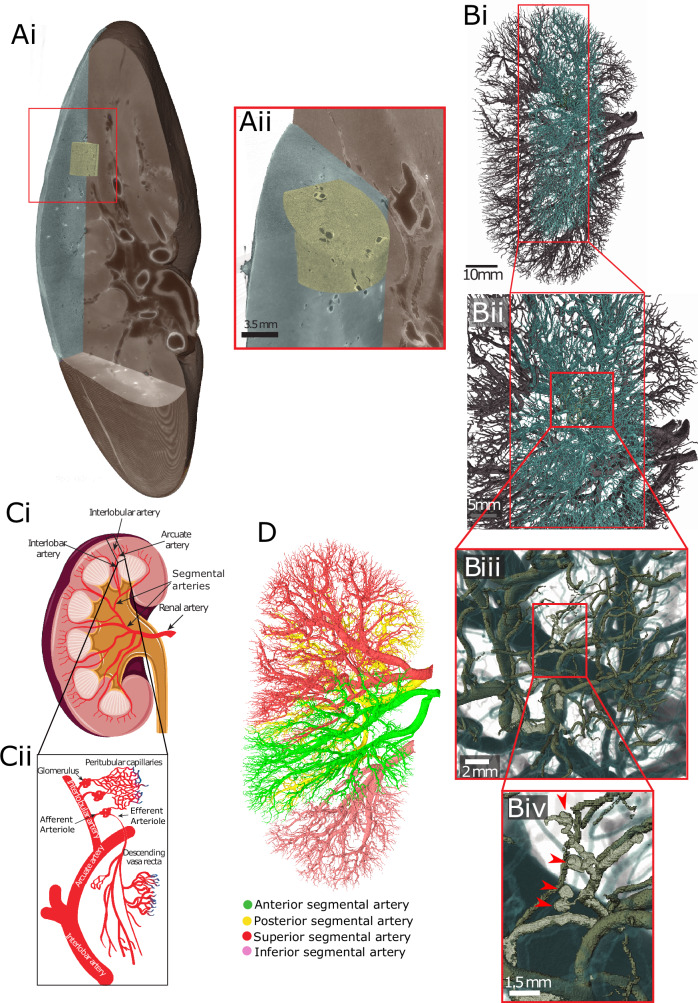
Multi-level segmentation of the human kidney arterial network. **A** Overview of the hierarchical image volumes that can be acquired with Hierarchical Phase-Contrast Tomography (HiP-CT). Brown, cyan and yellow volumes show the whole organ acquired at 25 µm per voxel, with sub-volumes (**Aii**) acquired at 6 and 2.6 µm per voxel, respectively, in the intact human kidney. **Bi**–**iv** Vascular segmentation performed across the three resolutions of HiP-CT data, enabling the intact arterial network to be visualised and segmented. Red arrows in (**Biv**) indicate segmented glomeruli. **Ci** Diagram of the anatomical organisation of the human kidney arterial network, with insert in (**Cii**) showing the smaller arterioles and capillaries. **D** The vasculature of the HiP-CT imaged kidney was partitioned into four territories, with each territory denoted by a different colour.

One imaging modality that could address the challenge of imaging intact organ vascular networks is synchrotron phase-contrast tomography. Hierarchical phase-contrast tomography (HiP-CT)^29^ is a technique which leverages the European Synchrotron Radiation Facility’s (ESRF) Extremely Brilliant Source (EBS), a high-energy fourth-generation synchrotron source, to image intact human organs. By utilising the high spatial coherence of the ESRF-EBS and the long beamlines available at ESRF, the development of HiP-CT^29^ has allowed the scaling of synchrotron phase-contrast tomography^30–32^ to sample sizes up to and including intact human organs. Datasets created with HiP-CT are hierarchically nested three-dimensional (3D) volumes at multiple resolutions, with exceptional soft tissue contrast spanning from small VOI to the whole intact organ (Fig. 1A). As an example of HiP-CT’s potential, we have previously profiled human glomerular morphology and number across cubic centimetres of intact human kidney^29^. However, the soft tissue contrast achievable with HiP-CT, coupled with its high spatial resolution, potentiates the visualisation and quantification of vascular networks across whole human organs, and could address the limitations of current imaging technologies used to map kidney vascular architecture.

Here, we demonstrate how the arterial network of an intact human kidney can be extracted and quantified across multiple length scales using HiP-CT without use of a vascular contrast agent. Our pipeline utilises the benefits of HiP-CT, such as validation of segmentation using multiscale data, whilst also providing solutions for the technical challenges associated with HiP-CT, for example, the collapse of large vessels. Within the human kidney, we delineated the extent and morphology of the vasculature, from the renal artery down to the interlobular arteries. In doing so, we were able to quantify heterogeneity in vascular architecture within the context of ordering schemes describing morphological network branching. We also demonstrate how the multiscale nature of HiP-CT allows estimation of the vascular network between the interlobular arteries and afferent arterioles in smaller VOIs, which we describe as 'local' scans,within the still-intact kidney. We perform a quantitative comparison between our human and previously published rodent kidney vascular networks, the latter of which has been used as inputs for biophysical modelling of kidney vascular blood flow^4–9^. We further demonstrate how the label-free nature and exceptional soft tissue contrast of HiP-CT allow vascular heterogeneity to be quantified in the context of other anatomical features, such as the different compartments of the kidney. Such spatial variations highlight the link between regional structure and function, reinforcing the importance of quantitative analyses for understanding and modelling regional microenvironments within the human kidney.

## Results

### HiP-CT can visualise the arterial vascular network in the intact human kidney

Using HiP-CT^29,33^ in a hierarchical fashion, we imaged the whole intact kidney obtained from a 63-year-old male organ donor. We initially performed an overview scan of the entire kidney at 25 μm per voxel, followed by selecting and imaging representative VOIs at 6.5 μm per voxel and 2.6 μm per voxel (Fig. 1A). As these image volumes are inherently aligned, expert annotation using renal anatomical landmarks (Fig. 1B) was applied to the image volumes taken at each resolution to produce a multiscale segmented model of the kidney’s arterial network (Supplementary Movie 1). From the segmented data, we were able to identify examples of and interconnect all known anatomical subdivisions of the kidney arterial system (Fig. 1C). The segmental pattern of anterior, posterior, superior and inferior territories supplying the kidney parenchyma was clearly delineated. Each vascular territory (Fig. 1D and Supplementary Movie 2) had a corresponding kidney arterial branch originating from the hilum, which bifurcated before hierarchical branching towards the cortical parenchyma.

### An image processing pipeline to reproducibly quantify the arterial network in human organs

**Fig. 2:**
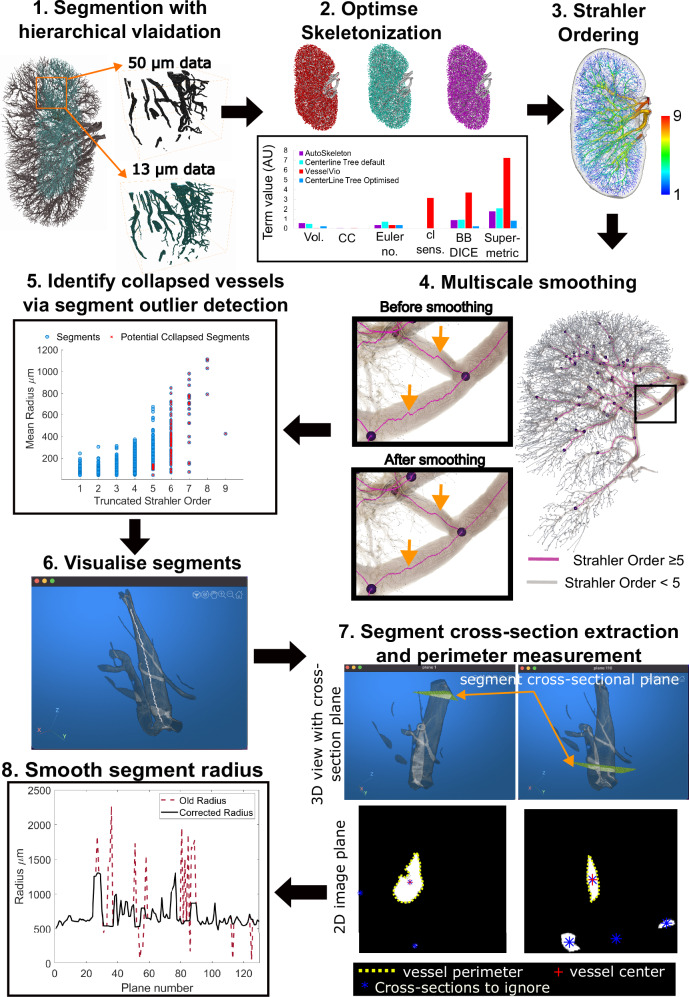
Overview of the pipeline for the extraction and correction of the human kidney vascular network skeleton. Step 1: Segmentation is performed with quantitative validation using a higher resolution VOI. Step 2: Skeletonization is optimised by comparison of skeletonization algorithms and the skeleton super-metric. The super-metric is a projection of the distance vector between the reconstructed skeleton and the segmented image onto a weighted space. It contains 5 contributing terms: network volume (Vol.), connected components (CC), Euler Number, Centerline sensitivity (cl sens.) and Bifurcation DICE (BB DICE). Step 3: An initial truncated Strahler order (tSO) calculation is made on the skeletonised network. Step 4: Using the tSO from Step 3 the network can be split into larger calibre (tSO $\ge 5$) and smaller calibre vessels (tSO $< 5$). The larger calibre vessel can then be smoothed as shown in insets, orange arrows show the points where smoothing has noticeably acted on regions of larger vessels. Step 5: tSO vs mean radius is plotted for every segment (blue circles); potential collapsed vessels (red crosses) are flagged for all larger vessels and identified in smaller calibre segments by those as having a radius below the 90% percentile for their tSO. Step 6: The segments identified as outliers are presented to an annotator in an interact pop-up window, which allows the annotator to visualise the segment and manually confirm if it should be corrected for collapse. Step 7: For vessels which are confirmed as collapsed, planes which are normal to the centreline of the vessel (indicated by orange arrows) are created at every point along the centreline, these are presented in pop-up windows to the annotator, as are the 2D image for each orthogonal plane (lower panels). From these 2D planes, the collapsed vessel is identified (red cross) and the perimeter (yellow dashed line) is extracted. The perimeter is used to calculate an equivalent radius and assigned as the new radius of the segment. Step 8: The new radii at each point are plotted, and outliers are removed to reduce the effect of any remaining tortuosity in the centerlines. At this stage, the annotator can also manually remove planes that are visibly affected by residual tortuosity.

We next sought to quantitate the arterial network in a reliable and reproducible manner. As we have previously shown that quantitative features of vascular networks can vary by the image processing pipeline used^6^, we developed our own bespoke image processing pipeline (Fig. 2), involving reduction of the initial HiP-CT image to a skeleton, or spatial graph representation, of the arterial network. The graph representation comprises a set of ‘nodes’; defined as 3D locations where vessels meet or end, and ‘segments’; defined as the connections between these nodes (see Supplementary Fig. 6A and Fig. 4A). Our pipeline comprises 8 steps, which are fully detailed in our Supplementary Note 2, and enables the generation of a spatial graph from segmented HiP-CT data, with quantification of error in segmentation (from multiscale comparison) and skeletonization (through application of the skeletonization metric).

Our pipeline first (Fig. 2, Step 1) assesses validation of the segmentation. By aligning segmentations from scans taken at 13 µm per voxel, with VOIs captured at 50 µm per voxel, the higher resolution scans served as ‘ground truth’ for the lower resolution scanning. We used the cl-DICE metric^34^ to quantify the overlapping vessel portions finding that 97% of vessels with a vessel lumen radius greater than 50 µm are detectable at 50 µm per voxel. Our next step (Fig. 2, Step 2) comprises the optimisation of the skeletonization algorithm. We applied three different skeletonization algorithms, and utilised the recently developed skeleton super-metric^6^ to determine the most suitable algorithm and its parameter optimisation. We found that the Centerline Tree algorithm (Amira-Avizo v2021.1) was the best candidate algorithm, as indicated by its lower super-metric value in comparison to other skeletonization algorithms (Fig. 2, Step 2). Thereafter, several steps were implemented to correct the skeleton for HiP-CT specific challenges (Fig. 2, Steps 3–8), namely the multiscale nature of the vasculature, and the presence of collapsed vessels as a consequence of the ex vivo, label-free HiP-CT protocol. The challenge of multiscale vascular trees was corrected using a truncated Strahler ordering system, which partitions the network into larger or smaller calibre vessels. Smoothing was then applied to all large calibre vessels to reduce tortuosity in the vessel centreline, an artefact which occurs due to the sensitivity of skeletonization algorithms to noise along the vessel surface (Fig. 2, Steps 3 and 4). Following this multiscale smoothing approach, Fig. 2 Steps 5 and 6 involved the identification and manual verification of collapsed vessels. Initially, all large-calibre vessels were flagged as potentially collapsed. Additionally, smaller calibre vessels that were potentially collapsed were identified based on their categorisation below the 10th percentile for radius in their truncated Strahler order (Fig. 2, Step 5). Once identified, collapsed vessels were subject to a bounding box, automatically extracted and thereafter manually determined whether correction of the radius was required to account for collapse (Fig. 2, Step 6). For vessels requiring correction, cross-sectional planes along the vessel centreline were extracted, and the radius was calculated based on the cross-sectional perimeter (Fig. 2, Step 6). Finally, the identification of outlier radii in these cross-sectional planes was performed, using a 95th and 5th percentile windowing for radius along the vessel length. Additionally, an option was applied to manually flag planes that appeared compromised by residual tortuosity in the vessel centreline (Fig. 2, Step 8).

The result of this novel pipeline, when applied to our HiP-CT data of human kidney, was the generation the first open-source spatial graph of the intact human kidney arterial vasculature, ranging from renal artery to interlobular arteries. We were able to identify 97% of vessels >50 µm radius across the whole intact human kidney. The final network consisted of 10,193 nodes, 376,603 points and 10,190 vessels. The total network volume was 1.68 × 10^12^ µm^3^, with a length of 2.3 × 10^7^ µm. This spatial graph, which is provided in our Supplementary Information, captures the morphological features and connectivity of the human kidney arterial vasculature, which was then used for downstream analyses as described below.

### Multiscale generational and ordering analysis of the arterial vasculature in the human kidney

**Fig. 3:**
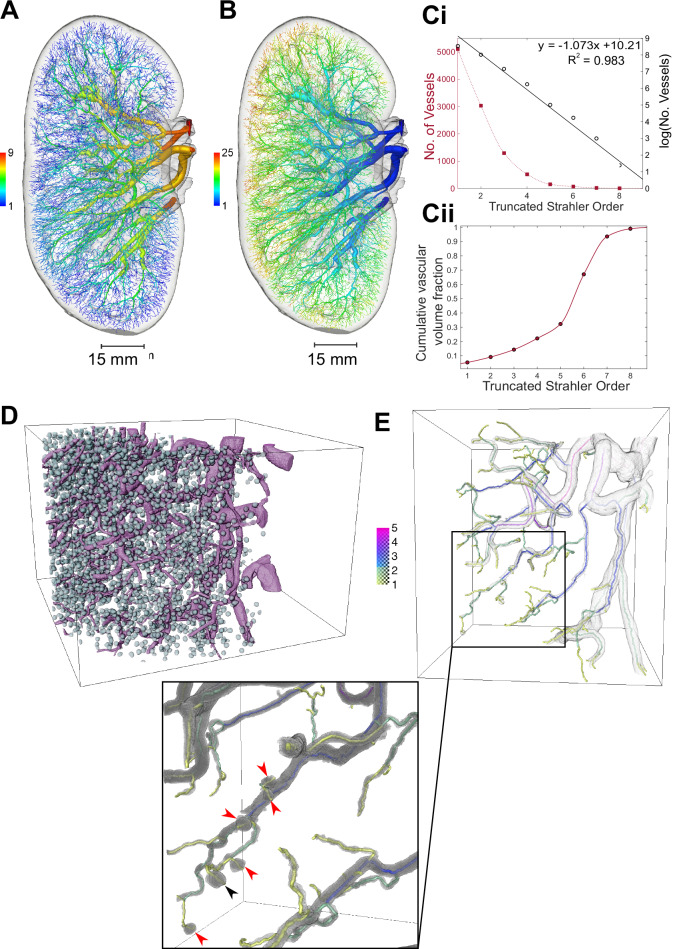
Ordering and branching ratio analyses of the human kidney vasculature. Rendering of the human kidney vascular network, with vessels coloured according to **A** Strahler order and **B** topological generation. **Ci** Plot showing the number of vessels per truncated Strahler order, with fit for the log plot to calculate branching ratio. **Cii** Truncated Strahler order against cumulative vascular volume fraction. **D** One of the VOIs with all glomeruli segmented. **E** A region within the VOI in (**D**) segmented at 2.6 µm per voxel, showing connection down to afferent glomeruli. Inset shows the six glomeruli that were connected back to the whole network. The five red arrows indicate glomeruli arising from non-terminal arteries, while the black arrowhead indicates a glomerulus arising from a terminal artery.

Having created a reproducible spatial graph of the human kidney arterial vasculature, we then performed topological generation^35^ and truncated Strahler ordering^36–38^ analyses. This resulted in nine truncated Strahler orders (Fig. 3A) and twenty-five topological generations (Fig. 3B). As the main artery supplying the kidney was cut during autopsy, we inferred that 10 truncated Strahler orders, representing 26 topological generations, were imaged over the intact human kidney with HiP-CT.

Strahler ordering, and other approaches to classify vascular networks, have potential caveats^39^ (See Supplementary Note 4), for example, the Strahler (or truncated Strahler) order of any individual vessel depends upon the downstream network, and thus on the identification of the network endpoint. Ideally, this endpoint would correspond to the afferent arteriole entering the renal glomerulus. However, at 50 µm per voxel resolution, we were unable to detect afferent arterioles, and thus, the smallest vessels, defined as truncated Strahler order 1 vessels, were the interlobular arteries.

Diameter-based statistical approaches to estimation of the Strahler order of the kidney vascular network’s terminal ends^11,38^ were not appropriate to correct for this due to the ex vivo and non-perfused nature of HiP-CT, as well as the connectivity of glomeruli relative to terminal vessel ends. Thus, we applied truncated Strahler ordering to our spatial graph and report how morphological features of vessels in the network vary with truncated Strahler order. We also mapped our truncated Strahler orders to known anatomical subdivisions of the arterial tree to give anatomical context. This resulted in the following classification: truncated Strahler orders 7–9 (*n* = 25 segments; mean radius = 929 ± 477 µm) mapped to the branches of the kidney artery entering the kidney hilum. Orders 5–6 comprised interlobar arteries (*n* = 219 segments; mean radius = 417 ± 247 µm), and orders 2–4 arcuate arteries (*n* = 4841 segments; mean radius = 78 ± 45 µm). Finally, interlobular arteries fell within orders 1–3 (*n* = 9430 segments; mean radius = 55 ± 23 µm). We further plotted the cumulative volume of the kidney vascular network (Fig. 3Cii), finding that over 20% of the volume of the network lies within Strahler orders 1–4, corresponding to segments from interlobular arteries and arcuate arteries. We found 5105 truncated Strahler order 1 segment and identified a logarithmic relationship between truncated Strahler order and segment number (Fig. 3Ci). Using this relationship, we determined the branching ratio within this subsection of the vascular tree to be 2.92, a value which is similar to that of the human pulmonary arterial tree (3.0^37^) and the rat kidney vasculature (2.85^11^).

To provide further context to the truncated Strahler order and to investigate the small-calibre vessels within the human kidney, we leveraged the hierarchical capability of HiP-CT. Using high-resolution VOIs, we segmented and counted all glomeruli within each of the 3 high-resolution VOIs of the HiP-CT data (Fig. 3D). We extrapolated from these VOIs to the total of ~1.2 million glomeruli in the intact kidney, which aligned well with estimates for adult males within a similar age range^40^. Given the 5105 truncated Strahler order 1 segments, the branching ratio of 2.921 and the total number of glomeruli, we estimated that there are a further 4–5 truncated Strahler orders between the end of our whole organ network and the afferent arterioles. To further evaluate this estimate, we assessed on high-resolution VOI, connecting six afferent arterioles of individual glomeruli back to the main vessel tree (Fig. 3E, Supplementary Movie 4 and Supplementary Fig. 8). Of the 6 glomeruli, 5 originated from non-terminal arteries and one from a terminal artery (Fig. 3E, red arrows and black arrows respectively). This supports recent findings^41^, in the rat kidney, which similarly demonstrated the existence of non-terminal branch arterioles, with potential contributions to the synchronicity of blood flow within the kidney^9,42^. The existence of non-terminal glomeruli also prevents the application of the statistical methods which have previously been used to estimate the true Strahler order of a truncated network. Given the presence of non-terminal glomeruli, statistical estimation of true Strahler order would necessitate connecting a larger number (~1000) of glomeruli back to the main tree to generate an accurate statistical representation of the proportion of terminal to non-terminal glomeruli. Such an estimation cannot be made with this dataset as, even with our highest resolution scans, the small vessels connecting the glomeruli to the main vascular tree could not be annotated reliably for a large number of cases. However, our estimation of total glomeruli number provides a data-driven estimate for the number of missing orders, and thus gives the context needed to support our use of the truncated Strahler order for our ongoing analysis.

### Analysis of human kidney vascular organisation in the context of Murray’s law and concordance with a rodent model organism

**Fig. 4:**
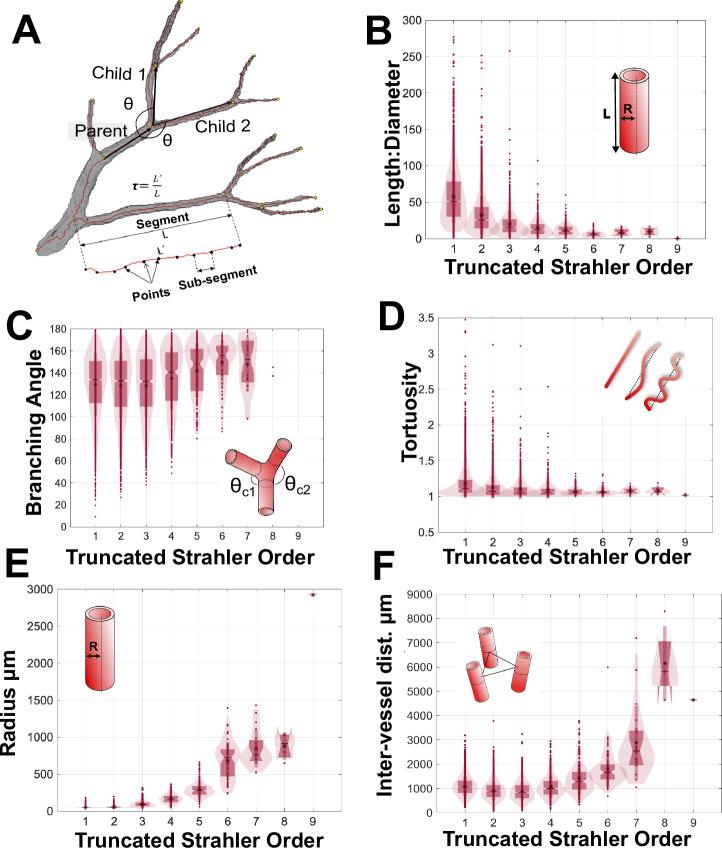
Quantitative branching metrics of the adult human kidney arterial network. **A** Schematic diagram of how the metrics in (**B**–**E**) are calculated. **B** The length:diameter ratio. **C** The branching angle between the child and parent segments. **D** The tortuosity of segments, **E** their radius, and **F** the inter-vessel distance as measured between the midpoint of each segment.

Vascular network geometric properties, including vessel diameters, lengths and branching angles, are key metrics for quantitative and objective comparison of vascular networks in health or disease^43,44^. Thus, we extracted and reported the metrics for the human kidney vasculature. Data were grouped according to truncated Strahler order (Fig. 4, Table 1) to enable quantitative comparison to rat and other human organ data. The raw data for each segment, which may serve as inputs for computational models, have been provided as Supplementary Information.

**Table 1:** Human kidney vascular branching metrics by truncated Strahler generation (means with standard deviation are shown)

Truncated Strahler order	No. of segments	Radium (µm)	Length µm × 10^3^	Tortuosity	Length:diameter	Vol. × 10^8^ µm^3^	Branching angle ^o^	IVD µm × 10^3^
1	5105	45 ± 5	2.6 ± 1.7	1.2 ± 0.20	57.4 ± 36	0.18 ± 0.22	129 ± 28	1.1 ± 0.4
2	3030	56 ± 15	1.8 ± 1.4	1.1 ± 0.16	32.3 ± 26	0.21 ± 0.46	128 ± 29	0.9 ± 0.4
3	1295	95 ± 37	1.8 ± 1.4	1.1 ± 0.14	20.2 ± 18	6.7 ± 10.3	128 ± 29	1.1 ± 0.4
4	516	165 ± 60	2.3 ± 1.9	1.1 ± 0.13	14.6 ± 12	25.5 ± 34.9	135 ± 29	1.4 ± 0.5
5	150	294 ± 110	3.3 ± 2.6	1.1 ± 0.05	12.2 ± 9.4	11.3 ± 17.1	142 ± 25	1.8 ± 0.7
6	69	684 ± 250	4.3 ± 3.1	1.1 ± 0.06	6.5 ± 4.5	84.6 ± 104	149 ± 21	2.9 ± 0.7
7	20	839 ± 251	7.2 ± 4.9	1.1 ± 0.05	8.5 ± 4.8	223 ± 302	148 ± 24	6.1 ± 1.5
8	4	877 ± 188	8.1 ± 4.7	1.1 ± 0.07	9.6 ± 6.3	227 ± 159	141 ± 6	4.7 ± 1.5
9	1	2923	669	1.0	0.2	180	-	-

Further quantitative analysis of the human kidney vascular network revealed that, as truncated Strahler order increased, there was a reduction in the ratio of vessel length:diameter (Fig. 4B). In contrast, the mean radius (Fig. 4E) and inter-vessel distance increased (Fig. 4F). Tortuosity did not vary significantly with truncated Strahler order (Fig. 4D); with most segments possessing tortuosity close to 1, thus implying limited deviation from a straight path. These findings are consistent with anticipated trends for a healthy tissue, wherein a vascular network is assumed to be a fractal structure, with branching pattern driven by optimised delivery of blood to the whole organ. Interestingly, within truncated Strahler orders 8–6, the mean branching angle was approximately 150°, decreasing to 130° for truncated Strahler orders 3–1 (Fig. 4C). Importantly, the latter value of 130° is the predicted optimal theoretical branching angle for volume-constrained vascular growth^45^.

**Fig. 5:**
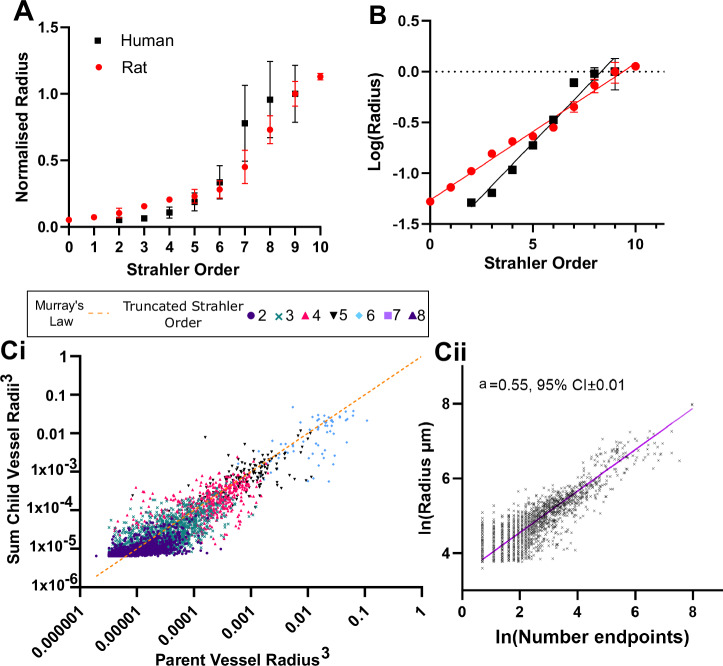
Comparison of human kidney vascular architecture to a rodent model and Murray’s theoretical law of energy balance. **A** Normalised radius against Strahler order for our data and for previously published rat kidney vascular data derived from Nordsletten et al.^11^. **B** The data are plotted for log(Radius), showing a similar pattern but with a significant statistical difference is found between the best fit for the two datasets. **Ci** Plot of Parent vessel cubed against Sum of cubed child vessels, Murray’s Law is shown in orange hatched line, points from each Truncated Strahler order are differentiated for clarity. **Cii** Plot of the log of the number of terminal downstream network ends against the radius for all segments in the network. The purple line shows the fit from the Standard Major axis regression, with the intercept a which is the radial scaling exponent and the 95% confidence intervals shown on the plot.

Simulation of kidney haemodynamics has previously been performed using μCT data from the rat kidney. To facilitate comparison between existing rat data and our human HiP-CT results, we aligned our network based on the Strahler order allocated to the segmental arteries, thus aligning Strahler order 9 in the previously published rat dataset^11^ to truncated Strahler order 8 of our human data. We then related normalised vessel metrics from each species, matching anatomically defined vessel types. The expected increase in vessel radius with order followed a similar trend between human and rat kidney (Fig. 5A). However human kidney vessel radii increased to a greater extent across Strahler orders than in the rat kidney, evaluated based on a fit of log(radius) to Strahler order (Fig. 5B), (*p* < 0.0001 Sum-of-F test F (DFn, DFd) = 700.6 (2, 12)). To provide additional insights into this difference observed between human and rat kidneys, we extracted radial scaling exponents of the human vascular network. The radial scaling exponent provides insight into how the network has structurally developed with respect to its functions such as the efficiency of blood flow and nutrient delivery to meet metabolic demands and minimise flow resistance^42,45,46^. Exponents of 0.33 and 0.5 each have theoretical bases in different models, (i) Murray’s law (expected exponent of 0.33 for all the whole network, derived from considering the energy balance between energy of flow and viscous drag), (ii) the West-Brown-Enquist (WBE) model which predicts 0.5 in larger vessels and 0.33 in smaller vessels, resulting from balancing the energy for metabolic distribution of blood across a fractal-like network. However, deviations from these exponent values and other variants of vascular scaling models have been widely reported^47–49^.

Previously in the rat kidney, Nordsletten et al. demonstrated a deviation from Murray’s law by ~1% for the rat kidney^11^. Figure 5Ci shows the cubed parent radius plotted against the cube sum of the child radii for our dataset, the theoretical Murray’s law is overlaid (orange hatched) to allow qualitative comparison to previous literature. To quantitatively evaluate whether our data are better represented by Murray’s law (expected radial scaling exponent 0.33) the WBE model (radial scaling exponent 0.33 in small and 0.5 in large vessels) or another model, we extracted the number of downstream terminal ends of the network for each vessel segment and the radius of that segment. Through a log-log plot of the data (Fig. 5Cii), the theoretical value of the exponent *a* was found to be 0.55. This value is higher than Murray’s law and closer to the WEB model and the values found in ref. ^49^ for the human pulmonary artery system.

### Regional heterogeneity within the kidney creates local microenvironments that potentiate specialised kidney functions

We then sought to compare heterogeneity in morphology of the human kidney vasculature according to anatomical regions within the human kidney, which may reflect specialised vascular functions. For example, the medulla of the kidney is predominantly vascularised by vasa recta; specialised capillaries which possess low oxygen tension. This configuration leads to physiological hypoxia that is inherent to the medulla’s urinary concentration mechanisms^50^. Further reflecting the importance of vascular morphology is the longstanding hypothesis, supported by blood oxygenation level-dependent MRI studies^51^, that vascular rarefaction in CKD results in hypoxia within the kidney cortex. In turn, this stimulates neighbouring cells into a pro-fibrotic phenotype, manifesting in replacement of normal kidney tissue by fibrosis and heralding loss of organ function^3^. Thus, regional heterogeneity of vascular morphology is fundamental for sustaining local microenvironmental features, such as hypoxia, that influence specialised organ functions. However, regional heterogeneity in vascular structure has not been quantitatively explored in the human kidney.

**Fig. 6:**
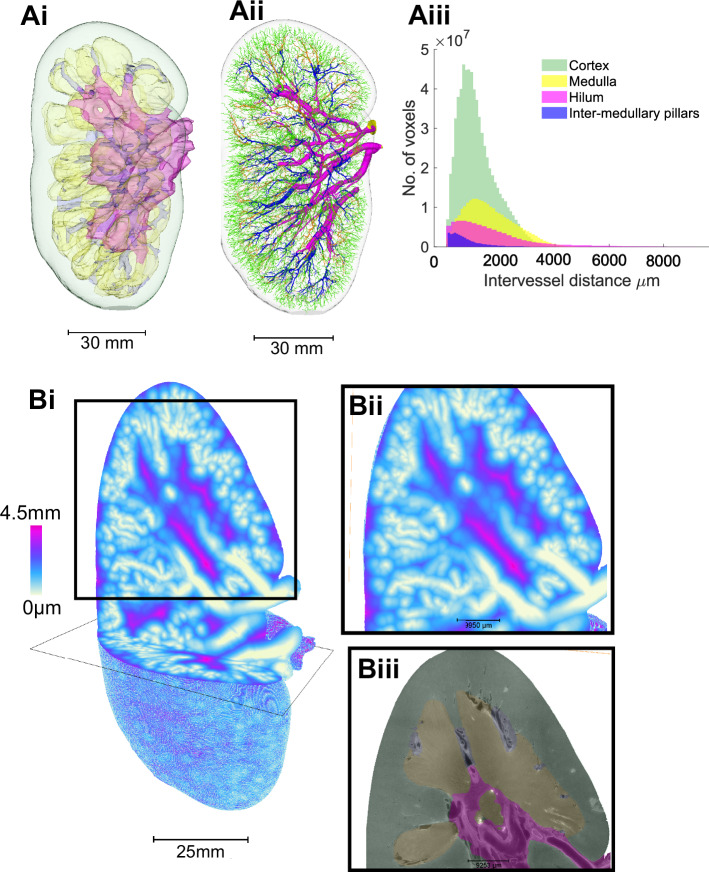
Analysis of compartmental heterogeneity in vascular branching metrics within the human kidney. **Ai** 3D surface masks of the kidney cortex (green), medulla (yellow) and hilum (pink), inter-medullar pillars (dark blue). **Aii** 3D reconstruction vasculature colour according to anatomical compartment within the human kidney cortex (green), medulla (yellow) and hilum (pink), inter-medullar pillars (dark blue). **Aiii** Inter-vessel distances are plotted against the total number of vessel voxels for each kidney compartment. **Bi** Visual heatmap of inter-vessel distance for the entire human kidney, where pink represents the largest inter-vessel distance (>4.5 mm) and white (0 mm) the smallest. **Bii** A digital zoomed region within cortex and medulla. **Biii** The 2D slice of the associated HiP-CT raw image with the compartments overlaid.

Leveraging the contrast-free approach of HiP-CT, we were able to segment the kidney into known anatomical compartments, including hilum, medulla, intramedullary kidney columns and cortex (Fig. 6Ai). We compartmentalised the vascular network according to these anatomical compartments (Fig. 6Aii). The total tissue volume of each compartment, in addition to the number of vessels, length, radius and volume of segmented vessels within each compartment, were quantified (Table 2). Most of the tissue volume of the human kidney was occupied by the cortex (63.7%) as compared with the medulla (23.5%), hilum (8.7%) or intermedullary pillars (4.1%). The number of segments of the vascular network within each compartment followed this trend. We then quantified (Fig. 6Aiii) and mapped (Fig. 6Bi–Biii) the inter-vessel distance, compartmentalised by hilum, medulla, cortex, and intermedullary pillars. Mean inter-vessel distances were calculated for each compartment, assessing the distribution of inter-vessel distance from the renal artery down to interlobular arteries (Table 2). The medulla had the highest inter-vessel distance. Whilst the cortex had a comparatively smaller inter-vessel distance than medulla and hilum, a large standard deviation for this value was noted within the cortex. This is illustrated by the heatmap in Fig. 6Bi, Bii, which identified small areas with inter-vessel distance >4.5 mm localised towards the kidney capsule.

**Table 2:** Human kidney vascular branching metrics compartmentalised by spatial zone with the organ

	Cortex	Medulla	Hilum	Intermedullary pillars	Organ
Volume of tissue, ×10^13^ µm^3^ (% of total)	8.70 (63.7%)	3.21 (23.5%)	1.18 (8.66%)	0.57 (4.14%)	13.7 (100%)
Number of segments* (% of total)	6141 (60.27%)	554 (5.4%)	151 (1.5%)	727 (7.1%)	10,190 (100%)
Mean segment length, µm ± STD	1999 ± 1374	1493 ± 1113	3993 ± 3568	1720 ± 1386	2260 ± 1720
Mean segment radius, µm ± STD	48 ± 12.6	95 ± 49	496 ± 335	136 ± 80	71 ± 87
Mean inter-vessel distances, ×10^3^ µm ± STD	1.10 ± 0.677	1.55 ± 0.881	1.55 ± 1.312	0.664 ± 0.543	1.2 ± 0.833
Mean segment volume, ×10^8^ µm^3^ ± STD	0.148 ± 0.116	0.623 ± 1.12	69.0 ± 147	1.73 ± 4.48	1.65 ± 20.6
Mean segment tortuosity ± STD	1.14 ± 0.17	1.08 ± 0.13	1.08 ± 0.1	1.1 ± 0.12	1.15 ± 0.18

*Segments that crossed over two regions were excluded.

## Discussion

Owing to the limited volume of tissue that can be imaged at high resolution using ex vivo 3D imaging modalities, such as μCT and lightsheet microscopy, and insufficient resolution of technologies routinely used in clinical practice, such as CT and MRI; it had previously been impractical to capture the vascular network of the intact adult human kidney beyond the very largest arteries. Here, using a synchrotron-phase contrast tomography technique, termed HiP-CT, we were able to image, segment and quantify the human kidney arterial network within an intact human kidney from renal artery to down to the level of interlobular arteries, without the need for exogenous contrast agents.

With HiP-CT, we show that vessels which have not been imaged in the intact human kidney previously, namely the interlobar to interlobular arteries, occupy approximately 20% of the arterial vascular volume of the organ. By imaging VOIs in the intact kidney at higher resolution, and aligning this with our lower resolution scans, we further demonstrate that, akin to rat^41^, and varying from the traditional hierarchy of the kidney vasculature observed in nephrology and anatomical textbooks, glomeruli in humans can originate from non-terminal arterioles. In further comparisons with the rat kidney vasculature, we found that although similar trends in vascular radius were seen, there was a significant difference in the change in radius with vessel order between species. This may be explained by the larger radii range in the human kidney between renal artery and afferent arterioles, relative to the volume of the human kidney; but could also be dependent on the difference in approach to calculation of the Strahler order for each study.

We also found that the exponent for radial scaling is closer to the WBE model (0.5) than Murray’s law value of 0.33. This is broadly in alignment with previous work^49^, where exponents of 0.47–0.58 were found for trees with vascular diameters ≥200 µm and 70–20≥ µm, respectively. Wide variation between theoretical exponents and those derived from real imaging data is widely accepted and often attributed to the complexity of real vessels including factors such as mechanical strain, the elastic nature of arteries during pulsatile flow and turbulent flow patterns^38,47,49,52,53^. Specific to the kidney, while Murray’s law or the WBE model assume idealised flow-optimised network, or ideal fractal scaling, kidneys likely exhibit non-optimal but functional scaling due to their high-resistance, low-compliance vascular network which is needed to support hemodynamic fluctuations due to changes in glomerular filtration and autoregulation^54^. However, it should also be noted that due to the ex vivo nature of HiP-CT, and the consequent lack of vascular tone, extracting such radial scaling laws from these data may have additional sources of error compared to in vivo imaging techniques.

Deviations from theoretical laws support the idea that vascular systems adapt based on tissue-specific demands rather than universal optimisation principles. This idea can be further supported by examining regional heterogeneity of vascular morphology in different anatomical zones of the kidney. The segmentation of hilar, medullary, intramedullary and cortical zones of HiP-CT images from the same kidney support this hypothesis. For example, the increased inter-vessel distance observed within the medulla, as compared to the cortex, is pertinent. The medulla experiences physiological hypoxia, and increased inter-vessel distance, paired with the oxygen diffusion limit, provides a potential anatomical rational for this phenomenon, in addition to the unique solute and gas exchange mechanisms that take part in this region of the kidney^50^. The data provided in this study, and resultant insights into how morphology of the kidney vasculature varies by different renal compartments, could shed light on the mechanisms underpinning the unique cellular and molecular adaptations of specialised endothelia across the kidney vascular network^2^. Our pipeline and the HiP-CT data provide a framework to potentially study how the vasculature within each anatomical compartments is differentially affected by kidney disease, with potential for understanding the basis of vascular rarefaction and pathological hypoxia^2,3^. Further studies with higher resolution HiP-CT or with microfill of the human kidney could potentially preserve vessel radius more accurately and resolve capillaries allowing extension of the work. Such information is important to acquire in human samples, as it could potentially influence simulations of haemodynamics, oxygenation or drug delivery^5,7^; and generation of synthetic vessel trees for in silico experiments^4,55^.

The human kidney vasculature is exquisitely specialised to meet the physiological demands of the kidney. Underpinning this specialisation is the cellular and molecular heterogeneity of endothelial beds within the renal vasculature^2^, of which we are gaining an increasing understanding due to the advent of improved techniques such as single-cell and spatially-resolved transcriptomics. The rapid and recent advances in our understanding of cellular and molecular heterogeneity of the kidney vasculature has not been matched by structural insights, likely because of limitations in imaging technologies. We have overcome many of these limitations using HiP-CT, where the exceptional contrast, coupled with appreciable spatial resolution at scale, allows us to capture and segment the 3D vascular architecture of an intact human kidney. Furthermore, within high-resolution VOIs, HiP-CT allows glomeruli and afferent arterioles to be segmented and, in selected cases, be connected back to the vascular tree of the intact whole organ.

Robust and reproducible analysis of vascular networks relies on the careful application of a multi-stage image processing pipeline, which we have outlined in this paper. We have developed an approach which utilises multiple annotators and comparison to higher resolution scans to validate segmentation accuracy as a crucial first step. Following segmentation, we have developed a skeletonisation approach, which can be scaled to large datasets, and also provides corrections for radius estimation when portions of the vasculature have collapsed. Finally, we applied a truncated Strahler ordering to the vessel spatial graph, providing a meaningful ordering system with respect to known anatomical vessel descriptions, as well as facilitating quantification of individual vessels within the vascular hierarchy. By developing and applying this pipeline, we have produced quantitative vascular branching metrics from an intact human organ for the first time. These metrics exceed other studies on cadaveric human kidney cast and dye injections, which report arterial branches corresponding to truncated Strahler orders 7–9^24,56^. We provide quantitative comparison between the human kidney vasculature and that of the rat, the latter of which has been key for inputs to generate biophysical models of kidney haemodynamics^9,11^.

The quantitative analysis pipeline performed in this paper serves multiple purposes. First, it allows the whole kidney vasculature dataset to be represented in a single spatial graph, comprising only kilobytes of data. This spatial graph, which is provided as Supplementary Data, is readily quantifiable. Whereas prior simulations of kidney haemodynamics and perfusion have relied on seminal μCT studies performed in rat, we provide, for the first time, a map of the kidney arterial network from renal artery to interlobular arteries. We demonstrated our segmentation approach to be accurate, with 97% of vessels of >50 µm radius captured across the intact human kidney. These data thus provide vital inputs for biophysical modelling of kidney physiology. The data also serves as a reference to study kidney diseases, in which vascular rarefaction is a pathophysiological hallmark^3^. The pipeline described could be used to generate vascular maps from multiple kidneys, or other human organs, potentiating spatial ‘atlases’ of human organ vasculature across healthy and pathological contexts. Beyond these, our openly available dataset has immediate practical applications, such as providing inputs for bioprinting and tissue engineering of artificial kidneys^57,58^ or planning surgical resection of kidney tumours whilst preserving kidney function^59^. These datasets can also be used as a tool for medical education and training, as well as for the creation and advancement of surgical methods.

There are several limitations of this work. Firstly, the low throughput of HiP-CT vascular segmentation warrants discussion. Here, we present the complete analysis from a single kidney as a framework for future studies of kidneys in health and disease or other intact human organs. The accuracy of the segmentation, however, lays a foundation for tools such as machine learning methods for automated segmentation of blood vasculature from imaging data^18,55,60,61^. HiP-CT imaging still cannot resolve afferent arteriole or capillary resolution across the whole organ, meaning that the contributions of peritubular capillaries or vasa recta are not incorporated. This also creates challenges for applying ordering schemes such as the Strahler order, where the true 0th order is the capillary bed. Previous approaches to estimating the distance of a terminal end in a truncated network from the capillary bed have relied on utilising diameter measurements of vessels to iteratively update the Strahler order of terminal ends^53^. This facilitates the creation of a connectivity matrix to estimate the downstream network^11,53^. However, diameter estimation is less accurate for HiP-CT, where vascular collapse makes radius estimates less consistent than, for example, when using microfill techniques. Using the high-resolution VOIs, we also demonstrated that glomeruli frequently emanate from non-terminal arterioles. Without connecting on the order of 1000s of glomeruli back to the main vascular tree as performed for small portions of rat kidney^9,41,42^, a connectivity matrix cannot be developed. However, the utility of any vascular classification scheme relies upon the ability to distinguish morphologically distinct vessel types, and to show logarithmic relations between morphology and classification orders^39^. Our truncated Strahler approach creates vessel orders which are able to separate morphologically distinct vessels (Supplementary Note 4), as well as demonstrating logarithmic relationships for radius and vessel number. We also found that truncated Strahler ordering also aligns well with the anatomically defined vessel classifications, as was the case for rat kidney^11^.

The future of HiP-CT and mapping the kidney vasculature is promising. The upcoming ESRF beamline (BM18) enables longer propagation distances than shown here, dramatically increasing the contrast sensitivity for the lower resolution scans. Further developments in scanning and data handling have already extended the capabilities of HiP-CT to create whole kidney overview datasets, with voxel sizes down to 9 µm/voxel, and to submicron voxel sizes in VOIs. Thus, future studies can leverage the greater detail available on low-resolution scans of the whole kidney, providing the potential to further assess phenomena such as the emergence of glomeruli from non-terminal arterioles, or potentially map entire organs down to the capillary level. As these developments unfold, we have created an open-access data portal (https://human-organ-atlas.esrf.eu/), enabling download and use of HiP-CT data by biomedical researchers across the world.

In summary, we have achieved quantitative mapping of the arterial network of an intact human kidney, from renal artery to interlobular arteries, for the first time. This vital step progresses our understanding of how physical properties of the kidney vasculature relate to cellular and molecular heterogeneity, whilst generating key inputs for future biophysical modelling of human kidney vascular physiology. Ultimately, we envisage that mapping of microstructural detail will become routine at the scale of the whole kidney, providing a means to link cellular events with organ physiology and pathology.

## Methods

### Sample preparation

An intact human kidney was obtained from a 63-year-old male (cause of death: pancreatic cancer), who consented to body donation to the Laboratoire d’Anatomie des Alpes Françaises before death. Transport and imaging protocols were approved by the French Health Ministry. Post mortem examination was conducted according to Quality Appraisal for Cadaveric Studies scale recommendations^33^. The body was embalmed by injecting 4500 mL of 1.15% formalin in lanolin, followed by 1.44% formalin, into the right carotid artery, before storage at 3.6 °C. During evisceration of the right kidney, vessels were exposed, and the surrounding fat and connective tissue were removed. The kidney was post-fixed in 4% neutral-buffered formaldehyde at room temperature for one week. The kidney was then dehydrated through an ethanol gradient over 9 days to a final equilibrium of 70%^33^. Each solution was four-fold greater than the volume of the organ, and, during dehydration, the solution was degassed using a diaphragm vacuum pump (Vacuubrand, MV2, 1.9m^3^/h) to remove excess dissolved gas. The dehydrated kidney was transferred to a polyethylene terephthalate jar where it was physically stabilised using a crushed agar-agar ethanol mixture, and then imaged^29,33^.

### Scanning, image acquisition and reconstruction

Imaging was performed on the BM05 beamline at the ESRF following the HiP-CT protocol^29,33^. Initially, the whole kidney was imaged at 25 µm per voxel (isotropic edge length)^62^. VOIs within the same kidney were also imaged at 6.5 and 2.6 µm per voxel^63–67^. Tomographic reconstruction was performed using the PyHST2 software^68^ and following the steps detailed in previous studies^29,33,34^. Briefly, a filtered back-projection algorithm, with single-distance phase retrieval, coupled to an unsharp mask filter, was applied to the collected radiographs. Reconstruction and scanning parameters are provided in Supplementary Note 1, Supplementary Tables 1 and 2. The reconstructed volumes were binned (averaged) to 50, 13, and 5.2 µm per voxel, respectively, to increase the signal-to-noise ratio, reduce inter-annotator variability and reduce computational load for subsequent image segmentation and quantification (see Supplementary Fig. 1). All reconstructed image volumes and metadata can be accessed at human-organ-atlas.esrf.eu. A table for direct DOI links for each dataset is provided in Supplementary Table 2.

### Image filtering, enhancement, and segmentation

Prior to manual segmentation, images were filtered to enhance blood vessel contrast using Amira-Avizo (v2021.1) software. A 3D median filter (iterations = 2 and 26 neighbourhood analysis) was used to reduce image noise. Image normalisation was performed using background detection correction (default parameter settings). A manual segmentation of the arterial networks was performed in Amira-Avizo using a combination of methodologies. First, a 3D region growing tool was used, where the user selects an initial voxel within a vessel lumen along with set intensity and contrast thresholds. Any voxel within the connected neighbourhood of the initially selected voxel with an intensity and contrast within thresholds are added to the region. Multiple seeds points and thresholds, as well as manual limits on the region, are used by the annotator to ensure the lumens of all vessels are accurately identified. In areas of collapsed or blood-filled vessels, annotators manually paint lumen voxels utilising three orthogonal views to ensure connection of the vascular network. An annotator continues this process in an iterative fashion by selecting seed points, altering the thresholds and manually correction, resulting in expansion of an interconnected vascular network (Method shown in Supplementary Movie 3). Once the first annotator has filled the interior of all vessels, data are passed to a second annotator, who repeats the process, but starting in reverse slice order. A third annotator serves as a proofreader by quantitatively reviewing the labels. The proofreader is presented with 5–9 randomised 2D slices of the data within any one of three orthogonal planes. They then count the number of vessels cross-sections present in the slice, recording the true positive and false negative number of vessel cross-sections that have been segmented. The proofreader returns the data to the initial two annotators, highlighting areas where vessels are not identified. This three-annotator process repeats iteratively until the proofreader does not find any false negatives. This method was applied to segment the kidney arterial network from the intact human kidney from the imaging data at 50 µm per voxel, and portions of the same network in the 13 and 5.2 µm per voxel datasets, approximately 250–300 h were needed to segment the kidney in this way.

A second approach to independently and quantitatively validate the segmentation of the lowest resolution data was performed using segmented VOIs of the higher resolution, 13 µm per voxel dataset. Here, the 13 µm per voxel VOIs were rigidly registered to the whole organ volume using the affine registration toolkit (Amira-Avizo) (See Supplementary Note 1.1, Supplementary Fig. 2 and Supplementary Tables 3 and 4). Overlapping portions of the 13 µm voxel segmentations and 50 µm per voxel datasets were extracted, and the 50 µm per voxel datasets were upsampled to the resolution of the 13 µm voxel dataset. An overlap measure, termed topological precision and recall score, following Paetzold et al.^34^, was applied (see Supplementary Note 2.1 and Supplementary Fig. 3).

### Skeletonization

To quantify branching metrics of the human kidney vasculature, the segmented 3D vascular network at 50 µm per voxel was skeletonised using the centreline tree algorithm in Amira-Avizo v2021.1. The choice of skeletonisation algorithm and the parameterising of the algorithm were optimised by utilising the super-metric approach, outlined by Walsh and Berg et al.^6^ (tube parameters: slope = 4 and zeroval = 10, see Supplementary Note 2.2 and Supplementary Fig. 4 for parameter optimisation results). The resulting spatial graph describes the vessel network in terms of ‘nodes’, ‘points’, ‘segments’, and ‘subsegments’. A segment is defined as being between a start and end node, corresponding to either a branching point leading into another segment branch, or a terminal end where no further branches were detectable. Between the start and terminal node of each segment lie subsegments with ‘points’, marking the start and end of each subsegment. Each subsegment has an associated radius and length (Supplementary Fig. 6A). A multiscale smoothing approach was applied to the larger vessels (those of Truncated Strahler order greater than 5). Iterative weighted smoothing was performed, where the smoothed location of any point is given by iteratively calculating weighted average of the current and two neighbour points. Parameter values were found empirically as 0.8, 0.1 and 15 for the neighbour points, current point and iterations, respectively. This reduced the tortuosity in the larger vessels (Fig. 2, Step 4), which occurs artefactually due to noise on the surface of the segmented large vessels, and which if uncorrected, impacts severely on the correction for collapsed radius vessels. Correction for collapsed vessels was delineated into two distinct cases. One case is a scenario in which there is a small collapsed portion in an otherwise patent vessel (Supplementary Fig. 5Bi, Bii). The second case applied when the majority of the vessel is collapsed (Supplementary Fig. 5Cii). The reduction in radius in the skeletonised form of the networks can be seen in Supplementary Fig. 5Bii, Cii. Correction for short subsegment collapsed vessels was performed by plotting radius along each segment. Subsegments where the radius was below the 5th percentile for that segment were replaced with the nearest neighbour. For larger collapsed vessels, the process is fully described in the “Results” section.

### Morphological analysis

Topological/morphological metrics of the network were calculated from the spatial graph as follows, with code provided at https://github.com/HiPCTProject/Skeleton_analysis:

i. *Branching angle* is calculated as either: (a) the angle between the two child segments from a common parent segment, or (b) the angle between a child segment and its parent segment. In both cases, the vector for the segment of parent and child were calculated between the start node and end node, irrespective of vascular tortuosity.
ii. *Tortuosity* is defined as the Euclidean distance between start and end node of a segment, divided by the sum of all subsegment lengths.
iii. *Radius* is calculated per segment as the mean of all subsegment radii. In cases where larger vessels had fully collapsed (See “Results” for details), the radius was defined as the equivalent radius for a circular vessel with the same length perimeter as the vessel cross-section in the binary image.
iv. *Length* is defined as the sum of all subsegment lengths.
v. *Inter-vessel distance* is calculated by two approaches to facilitate different analyses. First, using the segmentation binary image, the distance of every non-vessel voxel from its nearest vessel voxel was calculated *via* a 3D distance transform (ImageJ) applied to the binary vessel segmentation. Second, using the skeleton form, the Euclidean distance between the midpoint of every segment to its nearest-neighbouring segment midpoint was calculated.

In addition to the above metrics, we also assessed vessel generation, or order, using two methods. First, we used a variation of the centripetal system, known as the Strahler ordering system^36–38^, wherein the most distal, smallest segments are assigned as the first order. If two segments with the same order intersect, the resulting segment has one Strahler order greater. Alternatively, if two segments with different orders intersect, the higher order of the two is given to the resulting segment (Supplementary Fig. 6B). We used a variant of the Strahler order approach, which we term the truncated Strahler approach. Our 50 µm per voxel dataset does not provide sufficient resolution to image or segment down to afferent arterioles. Thus, the network created from this 50 µm per voxel dataset is truncated at the interlobular arteries. We assigned the terminal ends of our network as the first Strahler order, as opposed to applying statistical estimates to determine the Strahler order of these terminal ends based on diameter relative to the afferent arterioles, as performed previously^11,38^. Detailed discussion of this approach and alternative ordering approaches are discussed in the Supplementary Note 3. Second, we took a centrifugal, or ‘topological’ approach, starting with the most proximal artery as generation one. At each branching node the generation is increased, an approached which has been previously utilised^35^ (Supplementary Fig. 6C). From the ordering analyses, we assessed the branching ratio ($\gamma$) defined as the anti-log of the reciprocal for the linear fit to the plot of truncated Stahler order (*O*), against the logarithm of the number of segments (*N*) in each order:

1 $$N={N}_{0}{e}^{-\frac{O}{\gamma }}$$

The radius of the arterial network in the human kidney obtained from this study was compared to those of the rat kidney taken^11^, which was scanned with 20 and 4 µm per voxel using a microfilling approach.

### Radial scaling exponents

The radial scaling exponent for vascular networks refers to the relationship between the radii of parent and daughter vessels at a bifurcation. It is formulated as:

2 $${R}_{{parent}}^{1/a}=\sum _{i}{R}_{{child},i}^{1/a}$$

Where $a$ is the scaling exponent.

Murray’s Law^69^ describes an optimisation principle that minimises energy costs in blood flow by balancing viscous dissipation and metabolic maintenance, predicting a cubic relation, leading to a scaling exponent *a* = 0.33.

Murray’s law is derived from assuming minimal work in maintaining blood transport, leading to vessel radii scaling with the cube root of flow rate. Murray’s law also assumes, constant uniform metabolic demand of the tissue, laminar flow and that blood is a Newtonian fluid. In contrast, the West, Brown, and Enquist (WBE) model^70^ describes vascular networks as fractal-like structures that optimise metabolic energy distribution across the vascular network in its entirety. It predicts a 0.5 scaling exponent for large vessels and 0.33 for small vessels, accounting for hierarchical branching, where the emphasis is on efficiently delivering nutrients and waste exchange throughout the system.

Both models provide insights into vascular architecture but differ in scope, with real vascular networks often deviating due to biological variability and tissue-specific adaptations^47,49^.

Calculation of the radial exponent is done in this work following the regression-based method outlined in refs. ^48,49^. For each vessel in the network, the number of downstream endpoints of the network is counted. The radius and number of downstream tips are related by Eq 4. from ref. ^48^:

3 $${R}\alpha \,{N}_{d}^{a}$$

Where $R$= radius of a vessel in the network, ${N}_{d}$= the number of downstream endpoints from that vessel, $a$=the radial scaling exponent. Plotting the log-log relation of these two variables allows *a* to be estimated by regression analysis.

### Kidney compartment segmentation

Segmentation of the compartments within the human kidney, including cortex, medulla, intermedullary pillars and hilum, was performed in Dragonfly (version: 2021.3) using a 2D convolutional neural network (CNN). The final hyperparameters of the CNN are given in Supplementary Table 4. Correction of the CNN output was manually performed in by an expert in Amira-Avizo v2021.1 to provide the final compartment delineations. These compartments were used to group and then analyse vascular network parameters.

### Glomerular segmentation

40 3D patches (512 × 512 × 512) of the highest resolution data, captured at 2.6–5.6 µm per voxel, were extracted from multiple human kidneys scanned by HiP-CT, and the glomeruli were manually segmented. The widely utilised network nnU-net^71^ was trained using 35:5 cubes for a train:test split and a 70:30 training validation split. Training using 5-fold cross validation achieved a final DICE score of 0.928, 0.860, 0.906 for training, validation and test data, respectively. See Supplementary Note 3 for training results and nnU-net configuration. The plan files detailing all parameters for the training nnU-net are provided in Supplementary data. This trained network was used to perform inference of two VOIs of high-resolution data from the human kidney in this study, and count the number of glomeruli in each. Utilising the kidney anatomical compartment segmentation from above, the volume of cortical tissue within these high-resolution VOIs was calculated. For each VOI, the number of glomeruli and the volume of cortex in each VOI were used to estimate the total number of glomeruli in the entire kidney. Estimates of total glomerular number extrapolated to the entire kidney, from each VOI, were: 1.28 × 10^6^ and 1.12 × 10^6^ for VOI 3.1 and VOI 2.1, respectively.

### Statistical analysis

Statistical comparisons of vascular network morphology between human and rat kidney^11^ were performed in GraphPad Prism (version: 10.1.2). For all statistical tests, a *p*-value of less than 0.05 was considered statistically significant. In both the rat and human datasets, the segmental/feeding renal arteries were identified to be at Strahler orders 8 and truncated Strahler order 9, respectively. Radius against Strahler order were normalised to the 9th truncated Strahler order of the human data. Log of radius against truncated Strahler generation for the human kidney; and radius against Strahler Order of the rat kidney, were plotted facilitating a linear least squares regression analysis. A sum of squares *F* test was performed with the null hypothesis that a single set of global parameters for slope and intercept would fit vessel radius or vessel length for both the rat and human cases. For calculating the fit of the radial scaling exponent (*a*), we followed the approach of ref. ^48^ applying a Standard major axis regression to account for measurement error in both variables. This was performed in Matlab 2023a using the gmregress.m function with an alpha significance set to 0.05.

## Supplementary information

Supplementary Information_V11_reviewer_response

Supplementary_Movie 1

Supplementary_Movie 2

Supplementary_Movie 3

Supplementary_Movie 4

## Data Availability

The imaging data that form the basis of the study findings are freely available at the ESRF data repository (https://human-organ-atlas.esrf.eu) with links provided in Supplementary Note Supplementary Table 2. The spatial graph data of the kidney arterial network, along with the computed morphological parameters, can be accessed via links provided in Supplementary Note 7.

## Code availability

Codes for skeleton correction and topological analysis are available at https://github.com/HiPCTProject/Skeleton_analysis.
