# Supplementary material for: Mapping the arterial vascular network in an intact human kidney using hierarchical phase-contrast tomography
Source: Npj Imaging. 2025 Sep 3;3:39. doi: 10.1038/s44303-025-00090-2 (PMC12408821; doi:10.1038/s44303-025-00090-2)
Supplement: Supplementary file 1 — Supplementary Information_V11_reviewer_response [file 44303_2025_90_MOESM1_ESM.pdf]

Supplementary Information for: Mapping the arterial vascular network in an intact human kidney using hierarchical phase-contrast tomography

Running title: Analysis of the vasculature in an intact human kidney

Shahrokh Rahmani [1,2] , Daniyal J Jafree [3, 4, 5], Peter D Lee [1], Paul Tafforeau [6],Joseph Brunet[1,6] Sonal Nandanwar[1], Yang Zhou[1], Joseph Jacob [7,8], Alexandre Bellier [9], Maximilian Ackermann [10,11, 12] Danny D Jonigk [12,13], Rebecca J Shipley [1], David A Long [3,5], Claire L Walsh* [1]

1. Department of Mechanical Engineering, University College London, London, UK, WC1E 6BT.
2. National Heart & Lung Institute, Faculty of Medicine, Imperial College London, London, United Kingdom
3. Developmental Biology and Cancer Research & Teaching Department, UCL Great Ormond Street Institute of Child Health, University College London, London, UK, WC1N 1EH.
4. UCL MB/PhD Programme, Faculty of Medical Science, University College London, London, UK, WC1E 6BT.
5. UCL Centre of Kidney and Bladder Health, UCL London UK
6. European Synchrotron Radiation Facility, Grenoble, France, 38043.
7. Satsuma Lab, Centre for Medical Image Computing, UCL, London, UK.
8. Lungs for Living Research Centre, UCL, London, UK.
9. Department of Anatomy (LADAF), Grenoble Alpes University, Grenoble, France, 38058.
10. Institute of Anatomy, University Medical Center of the Johannes Gutenberg University Mainz, Mainz, Germany.
11. Institute of Pathology and Department of Molecular Pathology, Helios University Clinic Wuppertal, University of Witten-Herdecke, Wuppertal, Germany.
12. Institute of Pathology, RWTH Aachen Medical University, Aachen, Germany.
13. German Center for Lung Research (DZL), Biomedical Research in Endstage and Obstructive Lung Disease Hannover (BREATH), Hannover, Germany.

Contents

Table of Contents

Supplementary Note 1. Acquisition and Reconstruction Image 3

Supplementary Note 1.1. Registration of local zoom scans 6

Supplementary Note 2. Image Processing pipeline 8

Supplementary Note 2.1. Validation of segmentation using hierarchical data 8

Supplementary Note 2.2. Skeletonization optimisation 10

Supplementary Note 2.3. Correction of radius value for collapsed vessels 12

Supplementary Note 3. Glomerular Segmentation 14

Supplementary Note 4. Vessel Classification and Clustering approaches 18

Supplementary Note 5. Segmentation of compartments of human kidney 19

Supplementary Note 6. Supplementary Movie Legends 20

Supplementary Note 7. Supplementary Data 20

Supplementary Note 8. Supplementary References 22

Supplementary Note 1. Acquisition and Reconstruction Image

Basic parameters for each scan are provided in **Supplementary Table 1.** Reconstructed datasets can be obtained and downloaded from **human-organ-atlas.esrf.eu**, where they can also be viewed in-browser through neuroglancer. Metadata for each dataset, including scanning parameters, demographic and medical information for donors, as well as image reconstruction parameters, are available through this portal. Each dataset can be accessed and cited via a separate DOI, as listed in **Supplementary Table 2**.

Organ	Voxel size (μm)	Data label	Acquisition mode	Projection number	Propagation distance (m)	Attenuators	Average energy (keV)	Lateral field of view (mm)	Dose rate (Gy/s)	Scan time (min)
LADAF-2021-17-right-kidney	25.00	Complete organ	Half-acquisition	6000	3.5	Mo 0.1mm block, SiO2 32mm rods 8x4mm	~ 81	65.1	U.N	4.3
	6.5	VOI-02	Half-acquisition	6000	3.5	Mo 0.23mm block, SiO2 40mm rods 10x4mm	~ 83	25.0	U.N	U.N
	6.5	VOI-03	Half-acquisition	6000	3.5	Mo 0.23mm block, SiO2 40mm rods 10x4mm	~ 83	25.0	35	U.N
	2.6	VO-03.1	Half-acquisition	6000	1.4	Mo 0.23mm block, SiO2 40mm rods 10x4mm	~ 83	10.0	35	5.3
	2.6	VO-02.1	Half-acquisition	6000	1.4	Mo 0.23mm block, SiO2 40mm rods 10x4mm	~ 83	10.0	35	5.3

Supplementary Table 1 Scan Parameter.

Dataset name	***Voxel size (µm)***	***DOI***	***Link to interactive view***
25.0um_LADAF-2021-17_kidney-right_complete-organ	25	DOI10.15151/ESRF-DC-1773966439	https://human-organ-atlas.esrf.fr/datasets/1773966586
6.5um_LADAF-2021-17_kidney-right_VOI-03	6.5	http://doi.org/10.15151/ESRF-DC-2014789944	https://human-organ-atlas.esrf.fr/datasets/2016177220
6.5um_LADAF-2021-17_kidney-right_VOI-02	6.5	doi.org/10.15151/ESRF-DC-1773965451	https://human-organ-atlas.esrf.fr/datasets/1773965633
2.6um_LADAF-2021-17_kidney-right_VOI-03.1	2.6	http://doi.org/10.15151/ESRF-DC-2007535947	https://human-organ-atlas.esrf.fr/datasets/2008212318
6.5um_LADAF-2021-17_kidney-right_VOI-02.1	2.6	doi.org/10.15151/ESRF-DC-1773966217	https://human-organ-atlas.esrf.fr/datasets/1773965804

Supplementary Table 2 Dataset availability.

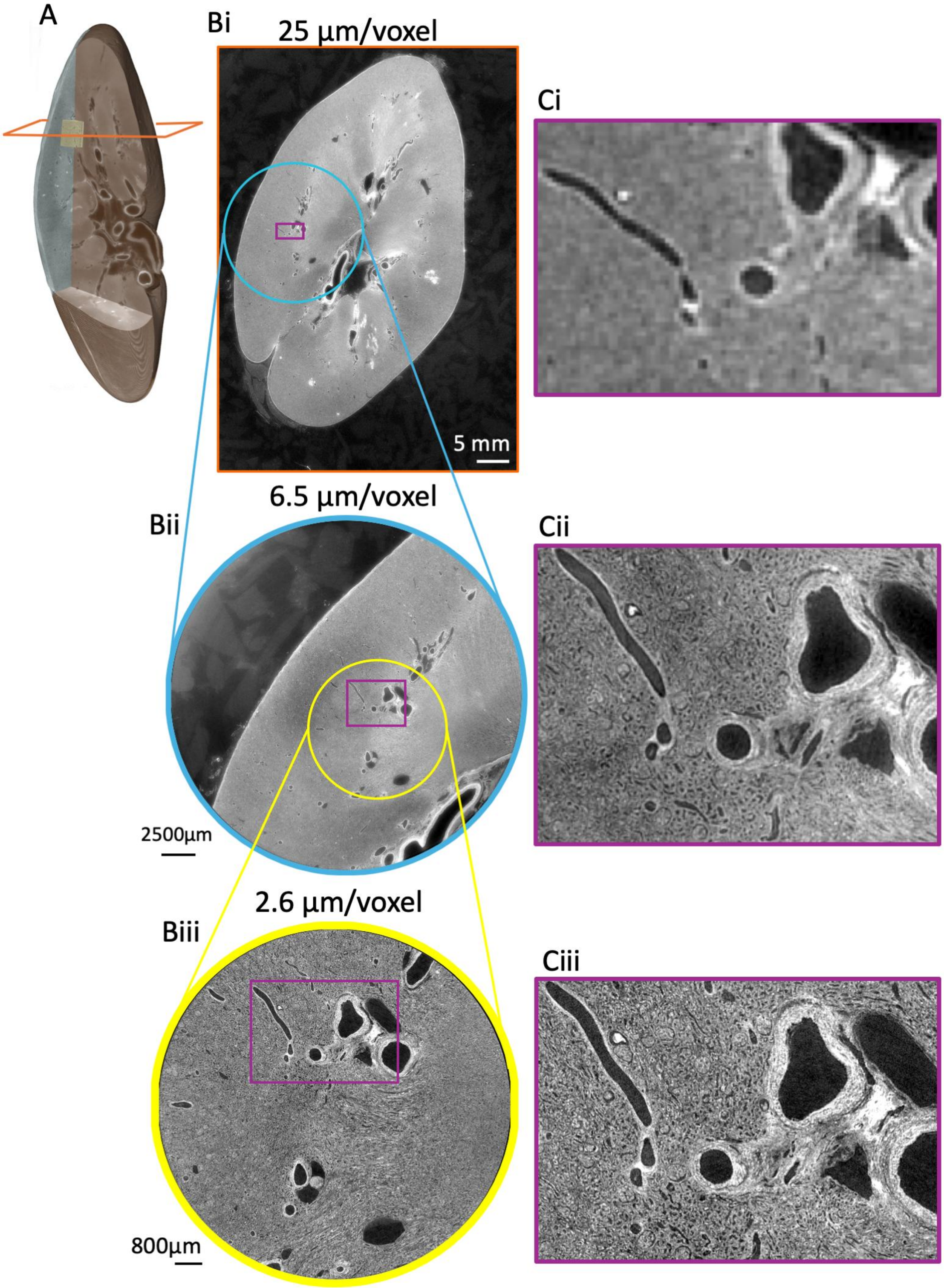

Supplementary Figure 1: The hierarchical HiP-CT imaging of the human kidney. **A**) An overview of all three nested images with orange square indicating the position of the xy slice in **Bi. Bi-Biii)** 25, 6.5 and 2.5 µm/voxel regions with digital zoom insets of the same regions in **Ci-Ciii)** showing the variation in level of detail at each resolution.

Supplementary Note 1.1. Registration of local zoom scans

The higher resolution volumes of interest (6.5 µm (down sampled to 13 µm) and 2.6 µm (downsampled to 5.2 µm)) were registered to the 25 µm per voxel (downsampled to 50 µm per voxel) image volume to facilitate annotation of vasculature across the scales. Image volumes were registered in in Amira-Avizo V2022.1 using mutual information metric, with rigid alignment and isotropic constraints. The initialisation of the volume positions was provided manually by the annotator. Parameter options are shown in **Supplementary Figure 2**

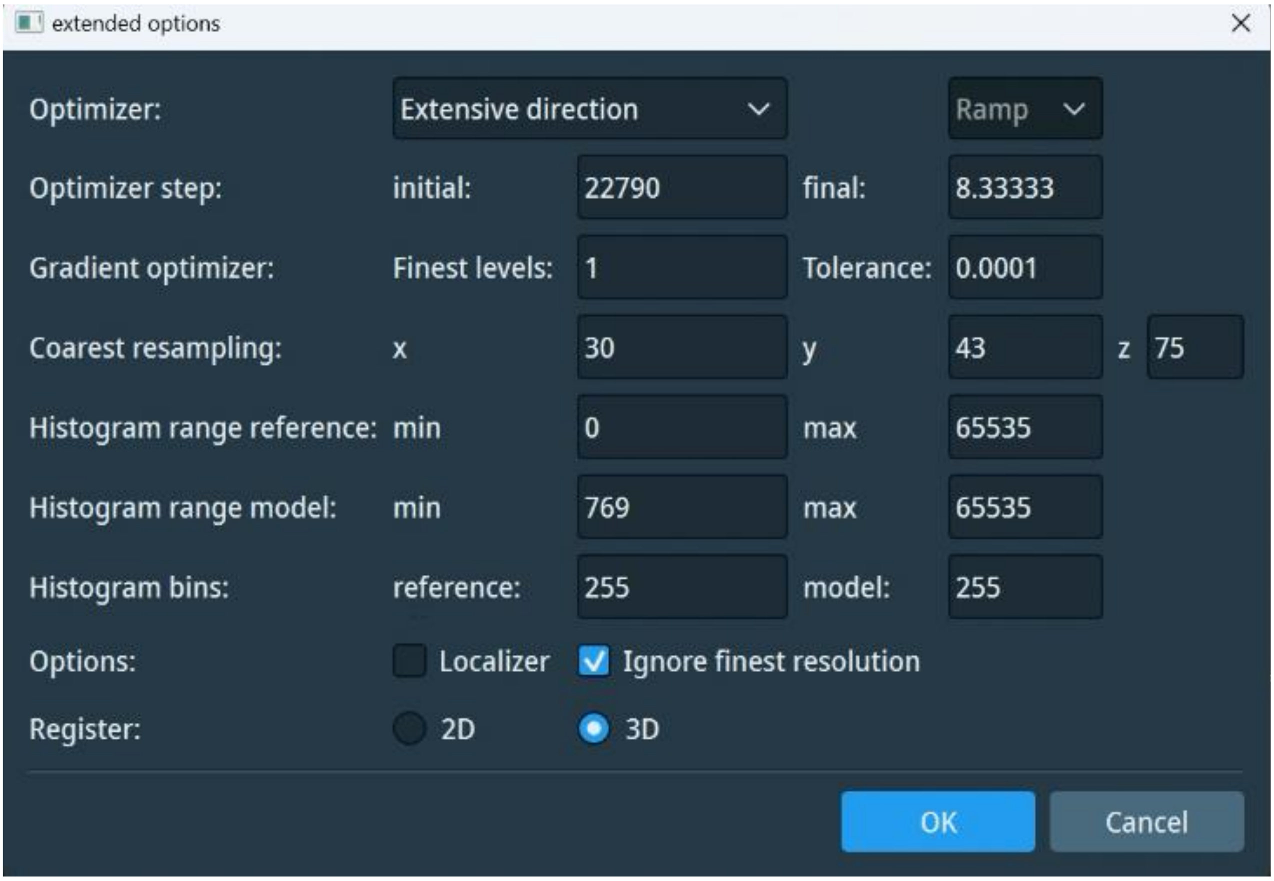

Supplementary Figure 2 Showing the parameter values used for registration of the high resolution volumes in to the overview whole kindey volume in Amira-Avizo V2022.2. The metric used was the mutual information, with constraints of ridgid transformation and Isotropic transformation applied.

The output of the registration – the transformation, can be re-applied to replicate the registered volume. This can be applied in Amira-Avizo or can be independently performed with the transformation is provided in **Supplementary Table 4.** This transformation is defined following the form of the Class SoTransform from the open inventor toolkit[1], where the geometric 3D

[1] https://www.openinventor.com/reference-manuals/NewRefMan1030/RefManJava/com/openinventor/inventor/nodes/SoTransform.html

transformation consists of in order: 1) a (possibly) non-uniform scale about an arbitrary point, 2) a rotation about an arbitrary point and axis, and 3) a translation. The transformations can considered be as being applied in that order. Matrices are pre-multiplied in the opposite order. e.g. where the transformation is applied from a combination of the translation matrix (T), the rotation matrix (R) and the scale matrix (S). The affine transformation M=S*R*T is calculated by applying scaling first, then the rotation and finally the translation. Each parameter is described in **Supplementary Table 3.**

Parameter name	Description	Default value
**translation**	Translation vector<br>Field containing a three-dimensional vector.	0 0 0
**rotation**	Rotation specification<br>4 values represent an axis of rotation followed by the amount of right-handed rotation about that axis, in degrees.	0 0 1 0
**scaleFactor**	Scale factors<br>Field containing a three-dimensional vector.	1 1 1
**scaleOrientation**	Rotational orientation for scale<br>Four floating point values separated by whitespace. The 4 values represent an axis of rotation followed by the amount of right-handed rotation about that axis, in degrees.	0 0 1 0
**center**	Origin for scale and rotation<br>Field containing a three-dimensional vector.	0 0 0

Supplementary Table 3. parameter descriptions to define the registered volume

Dataset	Voxel size	Translation	Rotation	Scale factor	Scale orientation	Centre
**2.6um_LADAF-2021-17_kidney-right_VOI-03.1**	5.2	6691.73, 24725.4, 28319.2	0.000967297 0.00446794, -0.99999 19.9447	1 1 1	0.00782034, -0.025613, -0.999641	5010.2 5010.2 3629.6
**6.5um_LADAF-2021-17_kidney-right_VOI-03**	13	-4068.46, 15889.5, 3561.3	-0.00163864, -0.0180448, -0.999836, 19.6033	1.00354 1.00354 1.00354	0.222067, 0.721417, -0.65593	12564.5 12207 38701

Supplementary Table 4. Registration parameters for the high-resolution volumes of interest to the overview whole organ image volume.

Supplementary Note 2. Image Processing pipeline

Supplementary Note 2.1. Validation of segmentation using hierarchical data

Quantification of the manual segmentation accuracy for the overview scans (down sampled to 50 µm per voxel) was provided by using segmentation of the 13 µm per voxel dataset (down sampled from 6.5 µm per voxel) as a 'ground truth', and using the cl-DICE metric [1] to quantify the difference. The cl-DICE metric quantifies the proportions of false positive and false negative vessels in 3D segmentations of vascular networks, by utilising the midline (or skeleton) representation of the segmentation. This reduces the bias for larger vessels to affect the metric score, as is the case for a classical DICE metric.

By using the 13 µm per voxel datasets as a 'ground truth' for the 50 µm per voxel dataset (**Supplementary Figure 3**), it is possible to visualise overlap between the segmentations for the

larger vessels, with additional smaller vessels present in the 13 µm per voxel segmentation that are not visible in the 50 µm per voxel segmentation. This is quantified by the topological precision (sensitive to false positives) and topological recall (sensitive to false negatives). The topological precision was found to be 0.97 and the topological recall was 0.5, with the undetected vessels noted to have a diameter less than 50 µm (**Supplementary Figure 3).** This demonstrates that our approach of manual annotation, utilising three independent annotators in an iterative manner, correctly identifies 97% of vessels >50 µm radius across the whole intact human kidney.

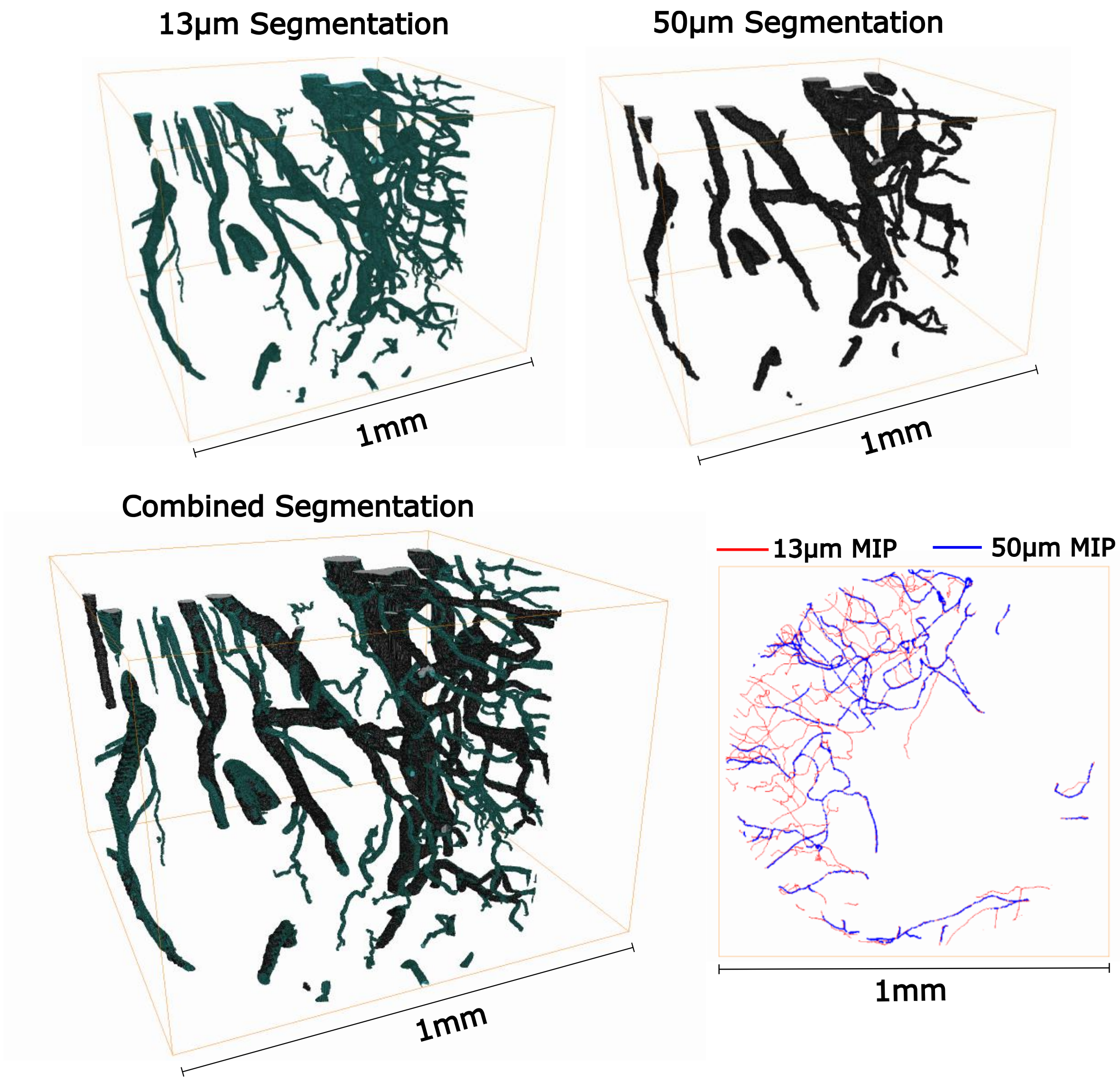

Supplementary Figure 3. Comparison of overlap between segmentations at 13 µm/voxel and 50 µm/voxel of aligned sub-volumes. Each sub volume is shown separately in the top row, the combined segmentations of both are overlaid in the lower left panel and overlay of the maximum intensity projects (MIP) through the z stack are shown in the lower right panel.

Supplementary Note 2.2. Skeletonization optimisation

Skeletonization reduces a binary image of a vascular network to a one-dimensional skeleton or spatial graph. This can be performed using several different algorithms, and thus the process is described and optimised as below. We optimised our skeletonisation following the approach of Walsh and Berg [2]. We considered three different skeletonization algorithms: Centerline-Tree (Amira-Avizo), AutoSkeleton (Amira-Avizo), and the Medial Thinning algorithm (implemented in VesselVio [3]). Firstly, to choose the most appropriate algorithm, we applied the recently defined skeleton super-metric [2] for each of the methods, which seeks to minimise the geometric difference between a validated segmentation and the skeleton representation. The super metric contains five components calculated relative to the binary segmentation. If I_0 is the binary image segmentation, we were able to define five features:

1. Total network volume V
2. Euler number of the largest connected component $\chi = N - E$
3. Number of connected components cc
4. The DICE score for the branching nodes in randomized sub volume of the network. B (range 0-1)
5. Cl-sensitivity score [1]. Cl (range 0-1)

These features were combined into a single function that can used as a cost function for skeleton optimisation. For any skeleton S_i created from a binary image I_0 we write this function as:

$$M_{S_i} = \sqrt{\left(\frac{V_{I_0}-V_{S_i}}{V_{I_0}}\right)^2 + \left(\frac{CC_{I_0}-CC_{S_i}}{CC_{I_0}}\right)^2 + \left(\frac{\chi_{I_0}-\chi_{S_i}}{\chi_{I_0}}\right)^2 + \left(\frac{B_{I_0}-B_{S_i}}{B_{I_0}}\right)^2 + \left(\frac{Cl_{I_0}-Cl_{S_i}}{Cl_{I_0}}\right)^2} \quad (1)$$

A skeleton with a super metric value of 0 can be considered optimal, and optimisation can be performed formally using, for examine, a Latin hyperparameter search, or informally with a random search approach from the user. In our case, this metric was used in a two phase process, firstly to choose skeletonization algorithms ,and secondly, to optimize the chosen algorithm. In the first phase, the Centreline Tree was compared to the Vesselvio and Autoskeleton algorithms, and was selected due to its low super-metric value, and as it is known to enforce tree topology and thus enable Strahler ordering analysis. Once selected, the two parameters of the Centreline Tree algorithm: slope and zeroVal, were optimized following [2] to find the optimal parameter values. The value for each component of the super-metric as well as the final metric for each of the initial skeletonization algorithms and for the optimized Centerline Tree algorithm is shown in **Supplementary Figure 4.**

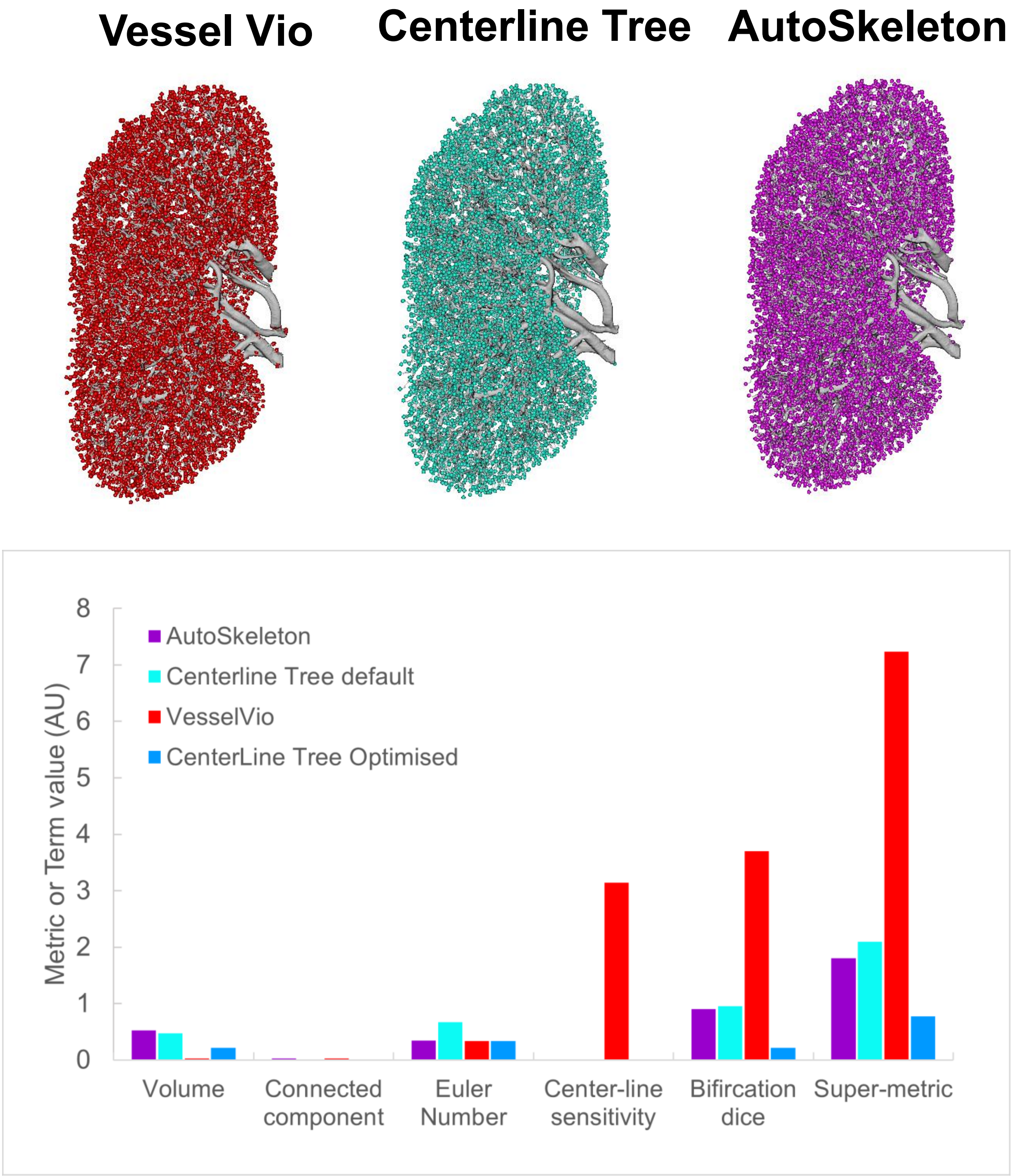

Supplementary Figure 4 Optimisation of the skeleton following segmentation. Top row. The output skeletons from the three algorithms used with their default parameter settings. Lower panel. Barchart showing the value of each component of the super metric as well as the final super-metric value for each skeletisation algorithm, and the optimised Centerline tree algorithm. Optimal values for each component and for the super-metric is zero.

Supplementary Note 2.3. Correction of radius value for collapsed vessels

A requirement for HiP-CT data is the correction of collapsed vessels. Some collapse of larger vessels during the HiP-CT preparation process is unavoidable (**Supplementary Figure 5** for examples). Whilst techniques such as microfill, could avoid this issue, microfill can lead to radius over estimation and would drastically increase sample preparation complexity. Additionally, microfill would interfere with the dynamic range sensitivity which is central to achieving such high contrast with HiP-CT. Thus, we chose to identify and correct the collapsed vessels in image post processing.

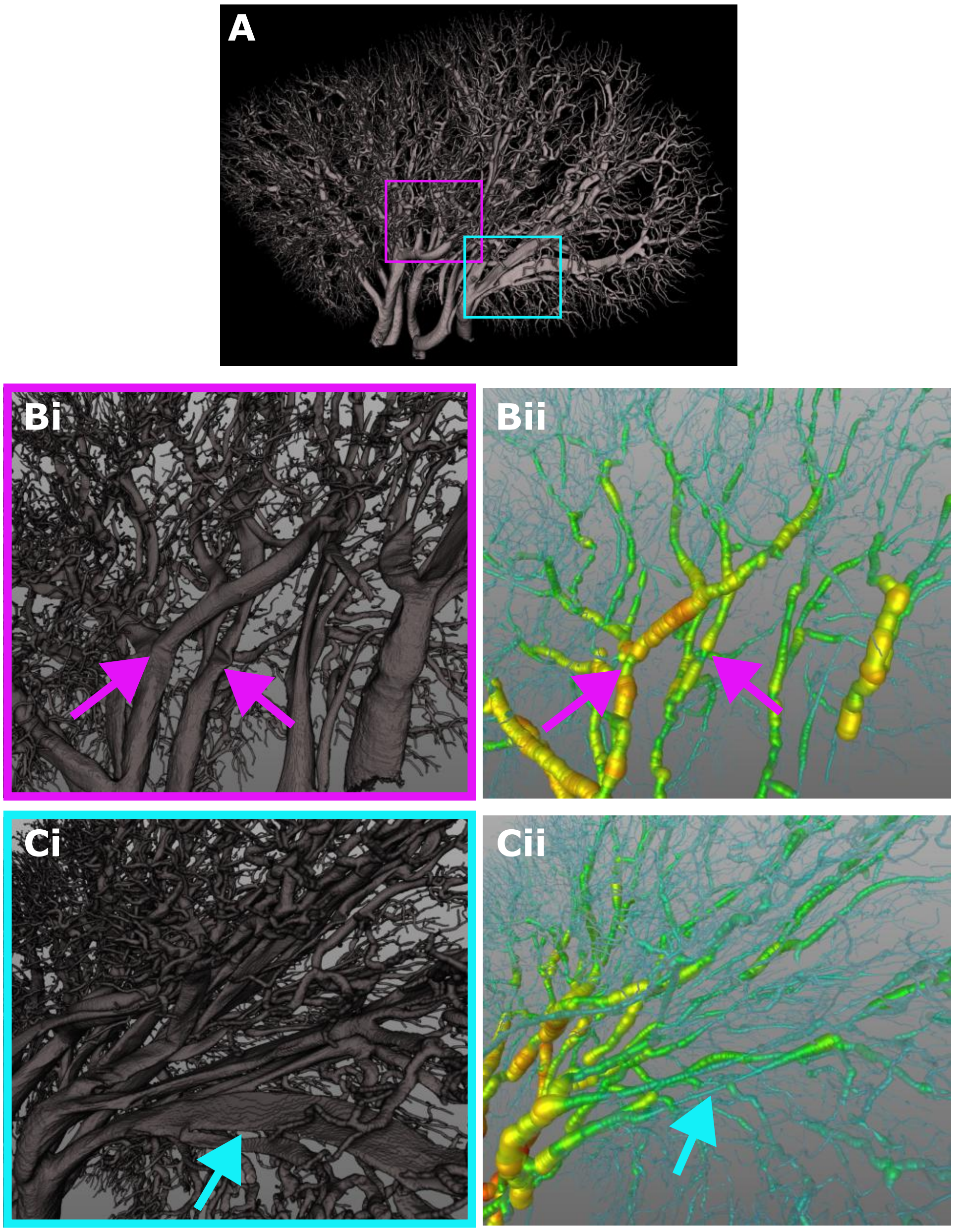

Supplementary Figure 5. Collapsed vessel types in HiP-CT. **A**) Overview image with regions of collapsed vessels identified by magenta and cyan squares. **Bi**) Example of two partially collapsed vessels (arrows). **Bii**) The same vessels in the spatial graph representation of the vessel network, with a decrease in vessel thickness clearly seen in both cases. **Ci**) Whole vessel collapse indicated by arrow. **Cii**) Same vessel shown in spatial graph format.

Collapsed vessels could be identified by visual inspection of the data in some cases (**Supplementary Figure 5A**), and are easily distinguished in the spatial graph due to the large underestimate of the radius in these segments **Supplementary Figure 5Cii**. We delineated two distinct cases of collapsed vessel, one where there is a small collapsed portion in an otherwise patent vessel (**Supplementary Figure 5Bi and Bii**), and secondly, where most of the vessel is collapsed (**Supplementary Figure 5Cii**). The reduction in radius in the skeletonised form of the networks can be seen in **Supplementary Figures 5Bii and 5Cii**.

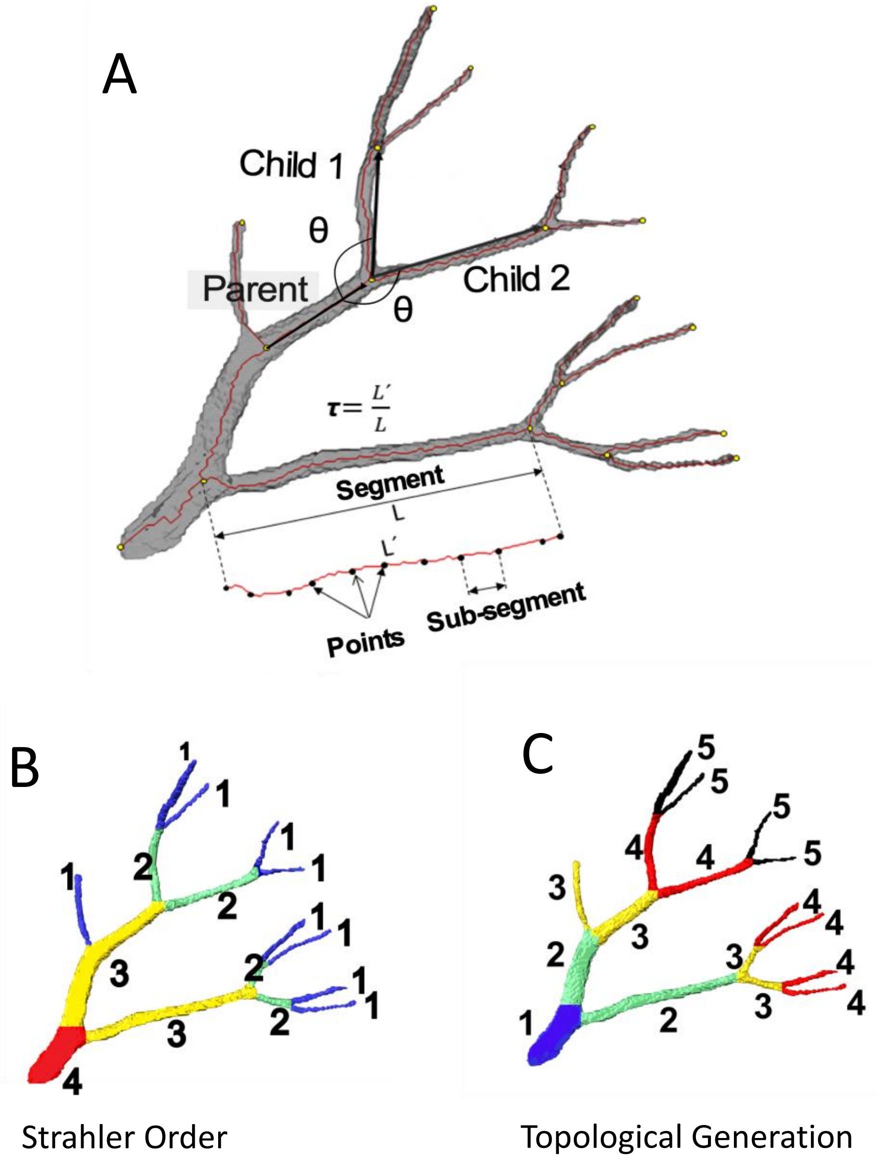

Supplementary Figure 6: Showing how metrics and ordering schema are calculated from a skeletonised network. A) Showing a partial vessel tree with nodes in yellow and the segments in red. The segment can is broken into sub-segments with points along. The diagram show how the tortuosity is calculated as well as how the parent child branching angles theta are defined. B) Show the Strahler ordering and C) the topological generation for the same network.

Supplementary Note 3. Glomerular Segmentation

The number of glomeruli in each of the high-resolution VOIs was estimated using the nnU-net convolutional neural network, with a configuration shown if **Supplementary Figure 7A.** This network was trained for 140 Epochs as shown in **Supplementary Figure 7B**. **Supplementary Supplementary Figure 7Bi** shows the over during training and **Supplementary Figure 7Bii** shows the mean DICE across 5-fold cross validation. Final DICE reached 0.928, and 0.906 for training and test data respectively. This trained network was used to perform inference across both the high-resolution columns, and the number of glomeruli counted. These were used to estimate the total number of glomeruli in the complete kidney. Values are provided in the table in **Supplementary Figure 7C.**

A

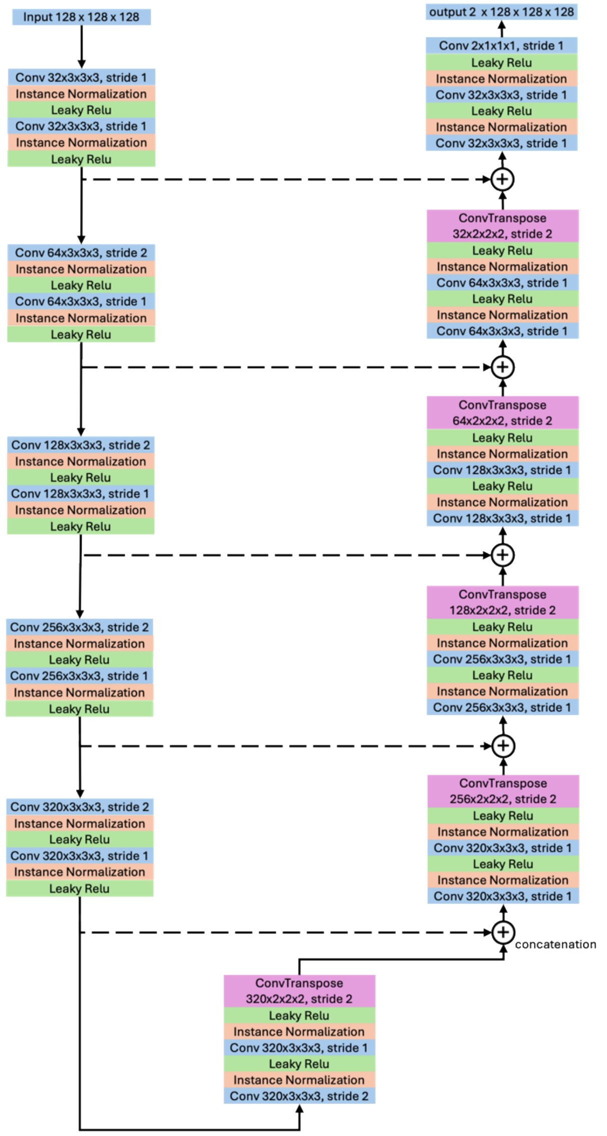

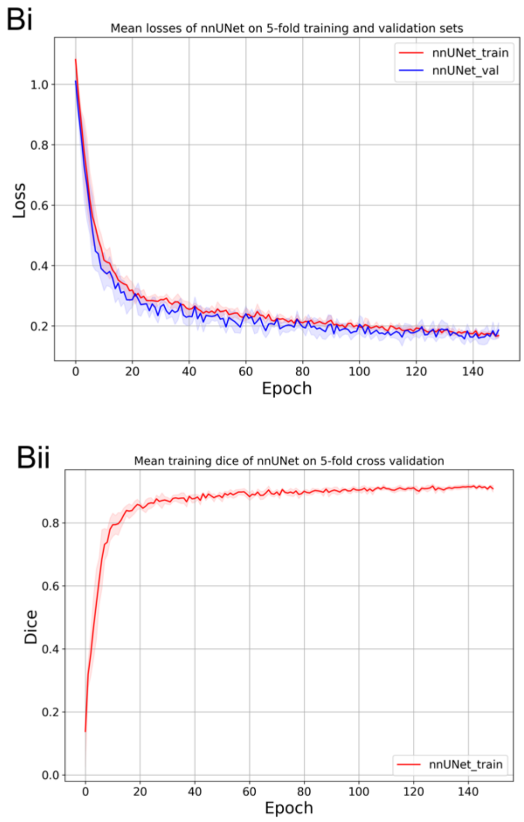

C

Scan	Number of glomeruli	Cortex mask (pixels)	Total no. of glomeruli
**VOI 3.1**	3093	13.6x10^6	1.12x10^6
**VOI 2.1**	10007	50.1x10^6	1.28 x10^6
**Whole kidney**	-	5.63x10^9	1.2x10^6

Supplementary Figure 7. A) showing the configuration of the U-net trained. Bi-Bii) showing the loss and dice respectively during the 140 epochs of training, each show the mean for 5 fold cross validation. C) Table providing the number for glomeruli number in each VOI and then the estimate in the whole kidney from these data.

Using the higher resolution HiP-CT VOIs, we were able to connect some of the segmented glomeruli back to the whole network (50 µm per voxel). **Supplementary Figure 8** show the same sub-region of the kidney segmented and skeletonised at 50, and 2.6 µm per voxel. At 2.6 µm per voxel it was possible to link six glomeruli back to the whole vascular network, and from there, show that this this created an additional two truncated Strahler orders between the end of the segmented whole vascular network and these glomeruli. Due to the resolution limit of our imaging, we could not connect more glomeruli back to the whole vasculature due to the fine diameter and tortuosity of these vessels, and thus we cannot at this stage calculate the number of glomeruli arising from terminal or non-terminal arteries.

However, we can provide an estimate based on the number of glomeruli, and the number of terminal ends in our network, that every truncated Strahler order 1 vessel within our fully connected network gives rise to approximately 235 glomeruli.

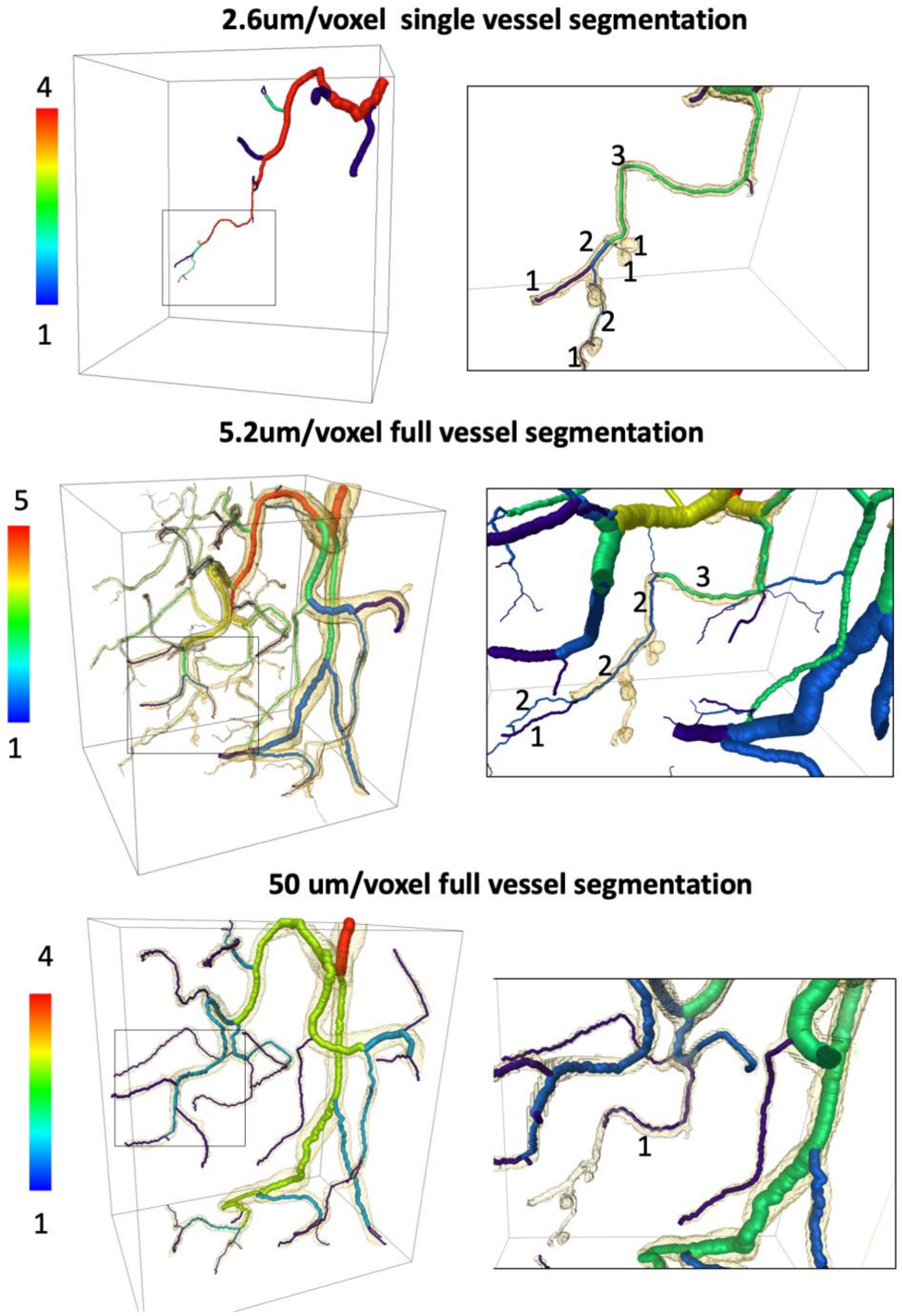

Supplementary Figure 8 Showing the same small Volume of interest (VOI) segmented and skeletonised at 2.6 μm/voxel for a single vessel down to 4 glomeruli (i.e. af ferent arterioles) this single vessel has four Strahler Orders. The same VOI was fully segmented at 5.2 μm/voxel (binned data), this showed that the same vessel segment contained 5 Strahler orders. When the skeleton for the single vessel at 2.6 μm/voxel is added to full skeleton at 5.2 μm/voxel the total Strahler re-performed 6 Strahler Orders can be counted, (without assuming any additional glomeruli at the terminal ends of the 5.6um dataset). The same VOI segmented fully at 50 μm/ voxel has 4 Strahler orders. Strahler orders are annotated on the insets for each case.

Supplementary Note 4. Vessel Classification and Clustering approaches

Vascular classification schemes have many varying approaches. In general, the goal of such analyses is to effectively separate different vessels into categories that facilitate either some anatomical understanding, or analysis of vessel architecture. The effectiveness of a scheme can be seen either by having non-ovelapping categories for vessels of the same 'type', or by having logarithmic relations between the classification approach and the morphological feature of interest. A diameter-ordered Strahler approach has been taken previously. This focuses on ensuring that vessels of the same diameter do not fall into different orders. i.e. that diameter ranges are non-overlapping for each order. Other approaches have been based on a variety of systems.
Vessel ordering schemes other than topological and Strahler can also be devised on the basis of these data. A particularly interesting approach is to allow vessel order to be assigned in an unsupervised way, based on the clustering of morphologically similar vessels to one another. k-means clustering is such an approach. **Supplementary Figure 9A** shows the output of applying k-means clustering to the segments of the network based on the structural features: length:diameter ratio, mean radius, inter-vessel distance (midpoint approach), topological generation, tortuosity and child parent branching angle. For k-means approaches, the number of clusters must be pre-specified, and this is typically done using multiple cluster value testing (with five replicates per number of clusters) and assessing the Davies-Bouldin Index for each number of clusters. A number of clusters from 3-25 were tested, and five was found to be optimal. **Supplementary Figure 9B** shows that each segment of the network could then be assigned to one of these clusters. **Supplementary Figure 9** show the output of the clustering for this kidney as well as the Davis-Bouldin Index for each cluster size and the David-Bouldin Index comparing Strahler Order, Topological Generation and K-means order **Supplementary Figure 9D**. Here we can see that the David-Bouldin Index for the Strahler and K-means are substantively lower than the topological equivalent, hence demonstrating that these two approaches are similar in their separation of distinct morphological clusters within the vessels of the human kidney vascular network.

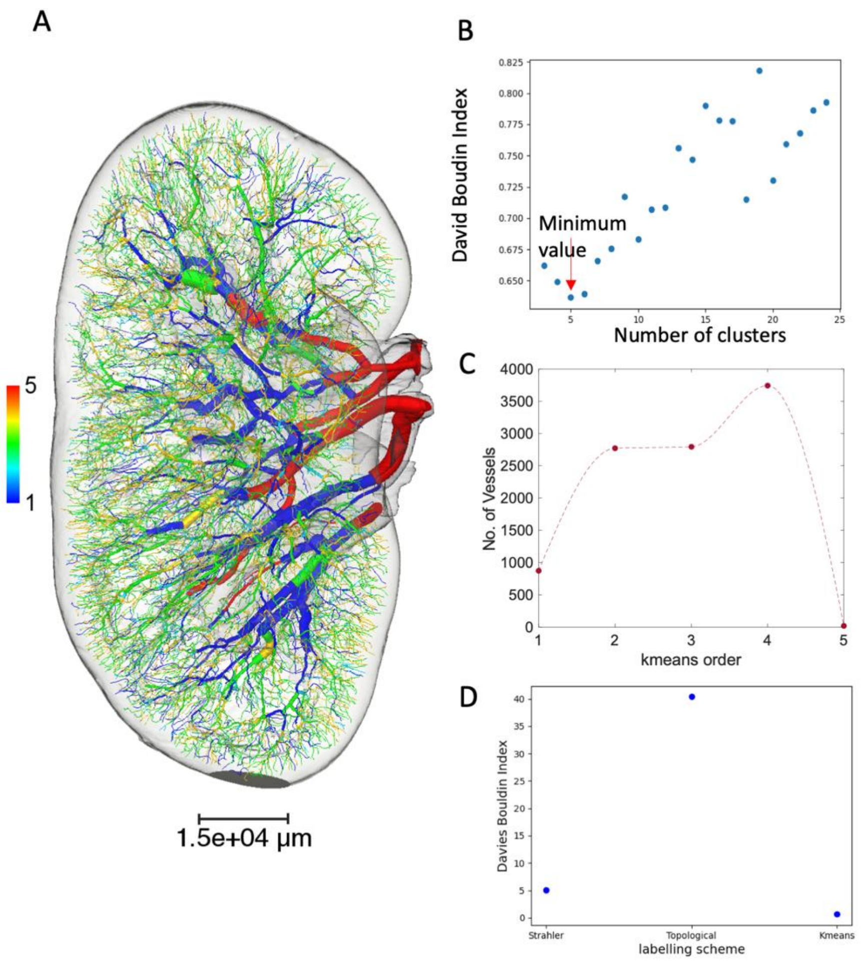

Supplementary Figure 9 K-means clustering approach. A) Kidney vessels coloured according to the k-means cluster. B) the David-Boudin (DB) index for multiple different cluster numbers with the red arrow indicating a minimum value of the index at 5 clusters. C) Shows the number of vessels per k-means Order, with 4 (yellow) vessels being the most prevalent and 5 red vessels being the fewest. C) shows the DB index for the three different Ordering schemes with the k means being the lowest indicating the most optimal number of parameters for representing the data variance.

Supplementary Note 5. Segmentation of compartments of human kidney

**Hyperparameter**	**Value**
First layer size	64
Layers	5
Patch size	64
Stride Ratio	0.5
Batch size	128

Epochs set	100
Epochs completed	63
Loss Function	Categorical Cross Entropy
Optimiser	AdaDelta
Augmentation	4 times
Augmentation types	Flip horz, Flip vert, rot, shear, scale (90-110%)
Learning rate	1
ρ	0.95
Epsilon	1.00E-07
Validation %	20%
Patience	20

Supplementary Table 5. Hyperparameters of the CNN used for segmentation of human kidney anatomical compartments

Supplementary Note 6. Supplementary Movie Legends

Supplementary movie 1. Multiscale segmentation of the intact human kidney, starting with the highest resolution VOI (5.2 µm per voxel) yellow where glomeruli can be seen, zooming out to the mid-level resolution (13 µm per voxel) cyan, and finally to the overview scan (50 µm per voxel) brown.

Supplementary movie 2. Overview segmentation with the four vascular territories differentiated. Anterior segmental artery (green), Posterior segmental artery(yellow), Superior segmental artery (red) inferior segmental artery (pink)

Supplementary movie 3. The process of performing semi-manual segmentation of the vascular lumen in Amira-Avizo using the magic wand tool.

Supplementary movie 4. Segmentation of the highest resolution VOI (5.2 µm per voxel) showing the segmentation and connection back to the vascular network of several glomeruli. Colours indicate the Truncated Strahler order for the small subsection of the network.

Supplementary Note 7. Supplementary Data

The complete kidney network is made up of three separate, unconnected graphs, referred to throughout the codes and data as cc1, cc4 and cc9. Each component is provided as a separate spatial graph with statistics. These spatial graphs can be used in accordance with the codes at https://github.com/HiPCTProject/Skeleton_analysis to reproduce our result. Owing to the size of the Supplementary data, all the below files can be found and downloaded from - **10.5281/zenodo.15275990,** the files are provided as a .zip and include:

File name	File type	Description
cc1_final_skeleton.attributegraph.am	amira spatial graph file	The spatial graph file for the first connected component of the kidney graph
cc1_final_skeleton_compartment_labelled.attributegraph.statistics.xml	Xml file	Statistics for the first connected component of the spatial graph with relation to the segmented anatomical compartments
cc1_final_skeleton.attributegraph.xml	Xml file	Xml providing the raw data for the nodes, segments and points of the spatial graph for the first connected component
cc4_final_skeleton.attributegraph.am	amira spatial graph file	The spatial graph file for the second connected component of the kidney graph
cc4_final_skeleton_compartment_labelled.attributegraph.statistics.xml	Xml file	Statistics for the second connected component of the spatial graph with relation to the segmented anatomical compartments
cc4_final_skeleton.attributegraph.xml	Xml file	Xml providing the raw data for the nodes, segments and points of the spatial graph for the second connected component
cc9_final_skeleton.attributegraph.am	amira spatial graph file	The spatial graph file for the third connected component of the kidney graph
cc9_final_skeleton_compartment_labelled.attributegraph.statistics.xml	Xml file	Statistics for the third connected component of the spatial graph with relation to the

		segmented anatomical compartments
cc9_final_skeleton.attributegraph.xml	Xml file	Xml providing the raw data for the nodes, segments and points of the spatial graph for the third connected component
Compartment.MaterialStatistics.xml	Xml file	Statistics for the anatomical compartments
Raw_segment_data_with_compartment.csv	csv	Segment data for the entire network (all three components) collated and with anatomical compartment ids.
Means_and_std_devs.xlsx	Xlsx	Excel detailing means and standard deviations used for comparison with rat data.
50um-LADAF-2021-17_labels_finalised_2_cc1.tif	3D tiff	The segmentation for the first connected component of the kidney
50um-LADAF-2021-17_labels_finalised_2_cc4.tif	3D tiff	The segmentation for the second connected component of the kidney
50um-LADAF-2021-17_labels_finalised_2_cc9.tif	3D tiff	The segmentation for the third connected component of the kidney
Plans.json	Json	Parameter script for nnU-net configuration

Supplementary Note 8. Supplementary References

[1] J. C. Paetzold *et al.*, "clDice—A novel connectivity-preserving loss function for vessel segmentation," in *Medical Imaging Meets NeurIPS 2019 Workshop*, 2019.

[2] C. L. Walsh, M. Berg, H. West, N. A. Holroyd, S. Walker-Samuel, and R. J. Shipley, “Reconstructing microvascular network skeletons from 3D images: what is the ground truth?,” *Comput Biol Med*, vol. 171, p. 108140, 2024.
[3] J. R. Bumgarner and R. J. Nelson, “Open-source analysis and visualization of segmented vasculature datasets with VesselVio,” *Cell Reports Methods*, vol. 2, no. 4, p. 100189, 2022, doi: https://doi.org/10.1016/j.crmeth.2022.100189.
